# Recent pace of change in human impact on the world’s ocean

**DOI:** 10.1038/s41598-019-47201-9

**Published:** 2019-08-12

**Authors:** Benjamin S. Halpern, Melanie Frazier, Jamie Afflerbach, Julia S. Lowndes, Fiorenza Micheli, Casey O’Hara, Courtney Scarborough, Kimberly A. Selkoe

**Affiliations:** 1 National Center for Ecological Analysis & Synthesis, University of California, 735 State St., Suite 300, Santa Barbara, CA 93101 USA; 2 Bren School of Environmental Science & Management, University of California, Santa Barbara, CA 93106 USA; 3 Stanford Center for Ocean Solutions, Pacific Grove, CA 93950 USA; 4 Hopkins Marine Station, Stanford University, Pacific Grove, CA 93950 USA

**Keywords:** Marine biology, Environmental impact

## Abstract

Humans interact with the oceans in diverse and profound ways. The scope, magnitude, footprint and ultimate cumulative impacts of human activities can threaten ocean ecosystems and have changed over time, resulting in new challenges and threats to marine ecosystems. A fundamental gap in understanding how humanity is affecting the oceans is our limited knowledge about the pace of change in cumulative impact on ocean ecosystems from expanding human activities – and the patterns, locations and drivers of most significant change. To help address this, we combined high resolution, annual data on the intensity of 14 human stressors and their impact on 21 marine ecosystems over 11 years (2003–2013) to assess pace of change in cumulative impacts on global oceans, where and how much that pace differs across the ocean, and which stressors and their impacts contribute most to those changes. We found that most of the ocean (59%) is experiencing significantly increasing cumulative impact, in particular due to climate change but also from fishing, land-based pollution and shipping. Nearly all countries saw increases in cumulative impacts in their coastal waters, as did all ecosystems, with coral reefs, seagrasses and mangroves at most risk. Mitigation of stressors most contributing to increases in overall cumulative impacts is urgently needed to sustain healthy oceans.

## Introduction

Impacts of human activities on the ocean have been shown to be substantial, ubiquitous^1^ and changing^2^. The resulting cumulative impact of these activities often leads to ecosystem degradation or even collapse^3–7^, and studies of individual marine ecosystems (e.g., coral reefs, kelp forests, seagrasses) have shown declines in condition globally due to increasing anthropogenic stressors^8–13^. Ongoing and emerging policy around managing for cumulative impacts to the oceans creates a pressing need to understand how, and how fast, cumulative impacts are changing. Expansion of existing uses of the ocean and emerging new ones – including offshore energy, ocean farming, and ocean mining – requires an understanding of what else is impacting those locations, how those new uses will add to existing impacts, and critically whether the cumulative impact of these ocean uses is changing, and how quickly. Both the European Union’s Marine Strategy Framework Directive and the United Nation**’**s Sustainable Development Goal 14 focus on assessing and reducing cumulative pressures to the oceans, and the upcoming renegotiation of the Aichi Biodiversity Targets in 2020 will benefit from a deeper understanding of the pace and pattern of change in cumulative impacts. Furthermore, the accelerating rate of creating marine protected areas (MPAs) to meet Convention on Biological Diversity (CBD) targets of 10% of the ocean within protected area by 2020 (ref.^14^), and the push globally to create very large MPAs^15^, could similarly benefit from detailed maps of where and how fast cumulative impacts are changing, as this information is critical to siting and managing effective protected areas.

To assess the pace of change in cumulative human impacts (CHI) we calculated and mapped the cumulative impact of 14 stressors related to human activities (including climate change, fishing, land-based pressures, and other commercial activities) on 21 different marine ecosystems globally for each of eleven years spanning 2003–2013 (Fig. S8), building on previous methods developed to calculate and map CHI^12,13^. The intensity of each stressor is mapped at 1 km resolution and rescaled to values between 0 and 1, using either known or estimated ecosystem thresholds or upper quantile values from the distribution of global stressor intensity values across years. The intensity of each stressor is then converted to an estimate of impact on each ecosystem by multiplying the stressor’s intensity by the corresponding ecosystem vulnerability where the ecosystem occurs^16^. The average impact of each stressor, across all ecosystems, is estimated by summing the stressor-by-ecosystem vulnerability combinations and dividing by the number of ecosystems within each cell. We then summed individual stressor impact scores for each cell to give the cumulative impact score (CHI, a unitless metric). We used per-cell linear regression across the 11 years of scores to determine magnitude, direction, and significance of change in CHI and mapped these changes globally at 1 km resolution. We further summarized the global results by ecosystem and country, focusing on the 3 nautical mile (nm) coastal area that humans most directly interact with and impact. All analyses were coded in R^17^. Full methodological details can be found in the Supplementary Information.

**Figure 1:**
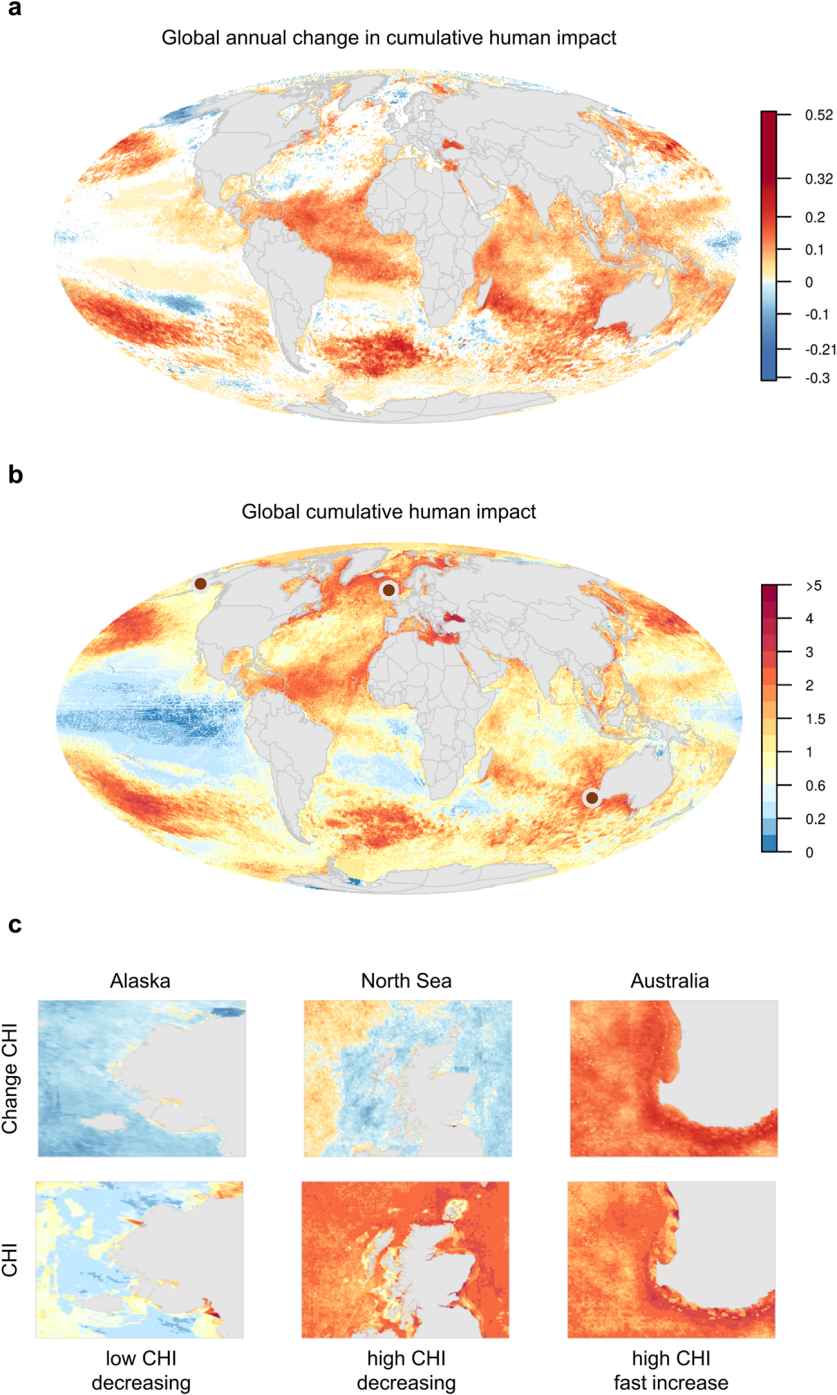
Global patterns of cumulative human impacts. (**a**) Annual change in CHI estimated using a linear regression model from 2003 to 2013 for each raster cell (white cells did not have a statistically significant trend, i.e., p > 0.05), Scale values reflect min and max raster values (−0.3 and 0.52) and 99.999^th^ quantile values (−0.21 and 0.32). (**b**) Static map of 2013 CHI. (C) Coastal areas displayed for both pace of change and 2013 CHI for regions (indicated by dots on global CHI map) with dominant patterns of: high CHI, fast increasing pace of change (Southwestern Australia); high CHI, decreasing (North Sea); and low CHI, decreasing (Yukon Delta region of Alaska). There were no clear examples of low CHI, fast increasing.

Cumulative impacts significantly increased (slope > 0, p < 0.05) for over half (59%) of the global ocean during an 11-year period from 2003–2013, and significantly decreased for only 5% of the ocean (Fig. 1a). Even in areas where change was small and non-significant, CHI values generally increased (total of 81% of the ocean; Fig. S6 shows pace of change without shading for significance). During this period, 15% of the ocean had CHI scores increase by >0.10 per year (Fig. S7). At this rate, regions with a CHI around 1.0 (the current global median, Table S6) will experience a doubling of impacts in about 10 years. Indeed, the median global cumulative impacts nearly doubled from 0.59 in 2003 to 1.0 in 2013 (Table S6). The fastest increases in CHI (>0.15 yr^−1^) occurred in about 3.6% of the ocean and included parts of the Black Sea, tropical Atlantic Ocean, temperate Northwest Pacific Ocean, and sub-tropical Indian, Atlantic, and Pacific Oceans.

**Figure 2:**
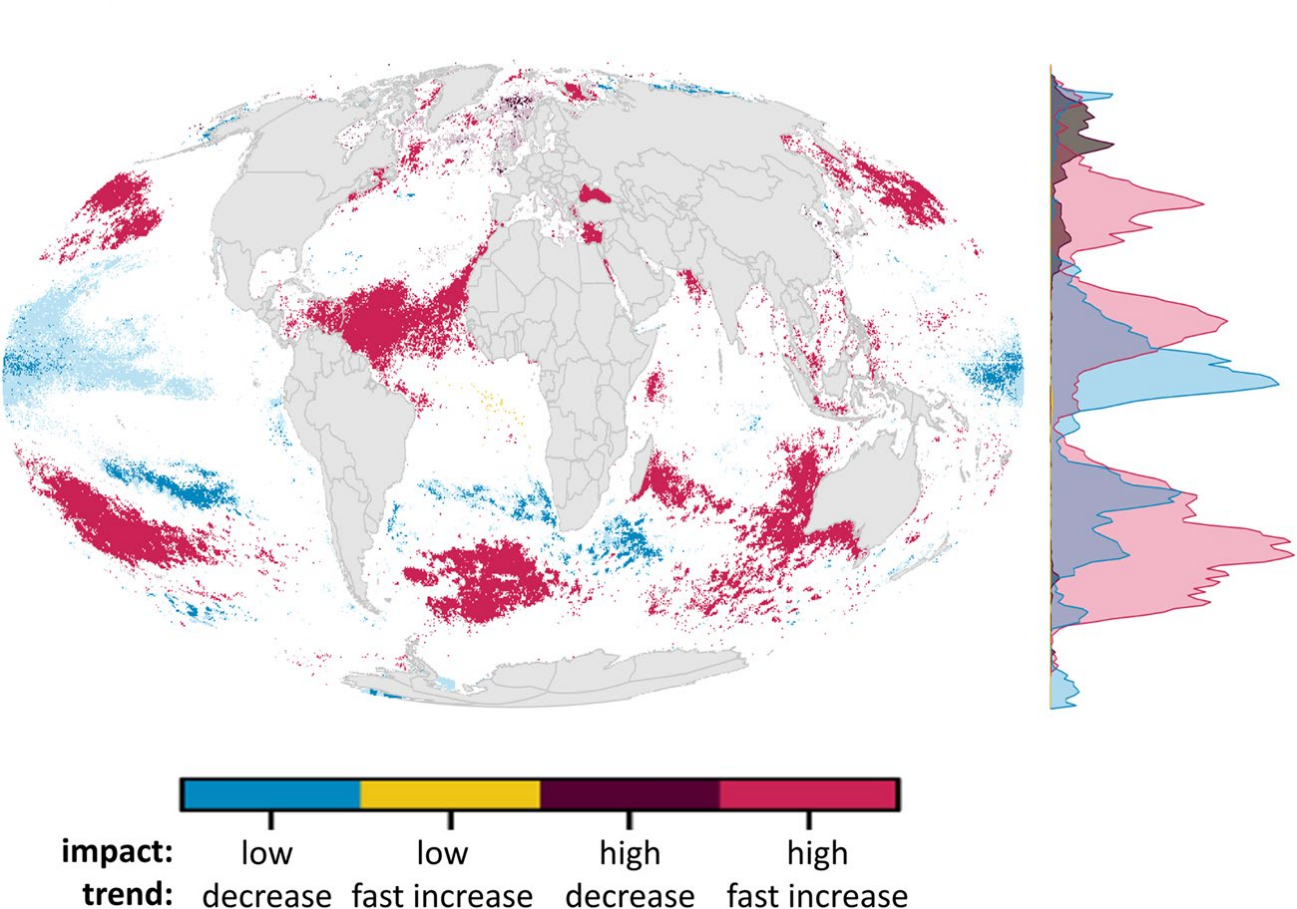
Overlap of extreme impact (high, low) and trend (fast increase, decrease) in cumulative impacts. Regions of low (<20^th^ quantile) and high (>80^th^ quantile) cumulative impact in 2013 were combined with regions where annual cumulative impacts were decreasing (trend ≤0, lighter colors represent non-significant trends) or quickly increasing (trend >80^th^ global quantile, corresponding to a slope estimate of 0.085, all significant trends). Trend estimates were calculated using a linear regression model from 2003–2013 cumulative human impacts. Density plot on right shows the distribution of values across latitude.

**Figure 3:**
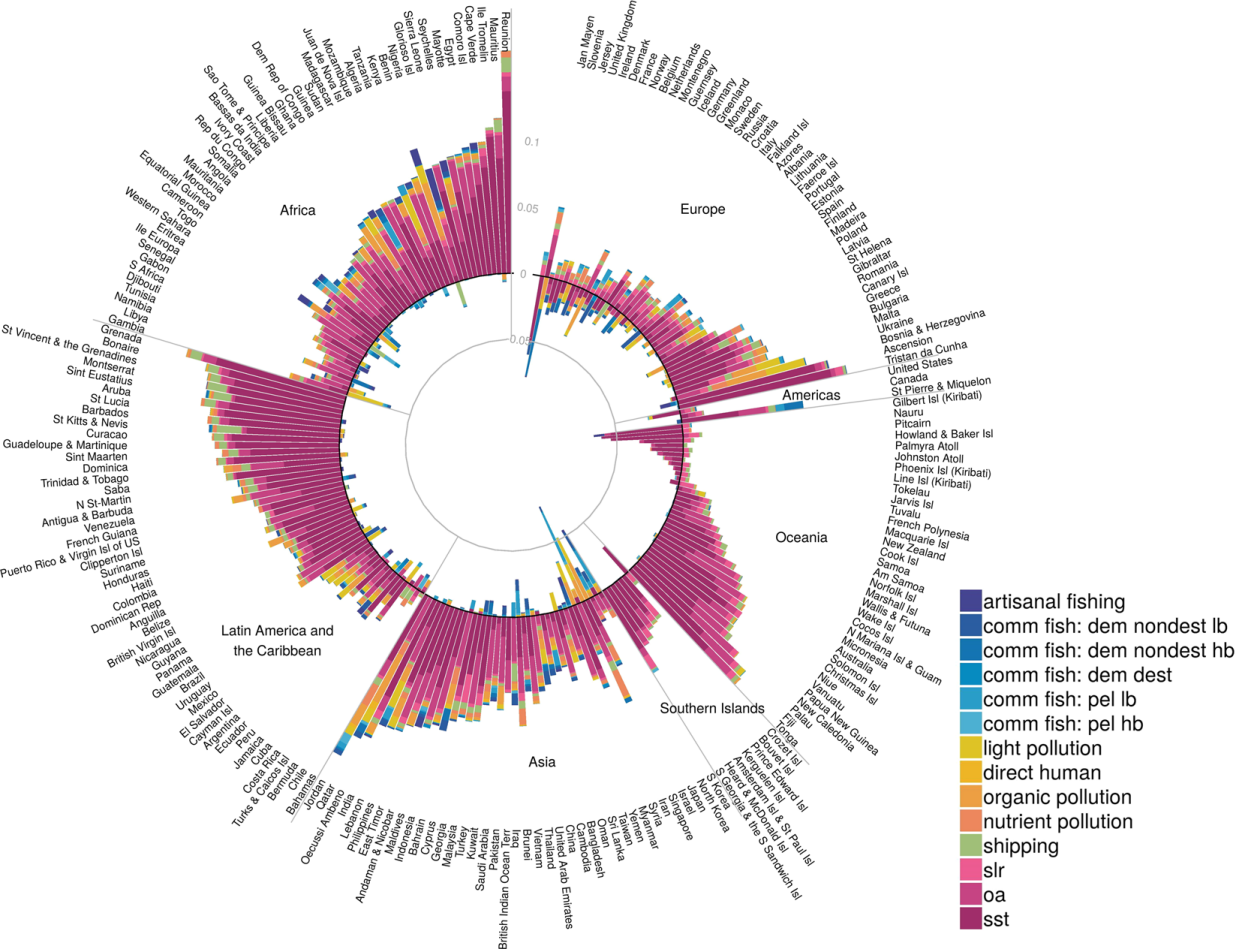
Annual pace of change in CHI per year in all 14 impacts comprising the cumulative impacts within coastal (0–3 nm) regions of each country’s EEZ. Outer bars greater than zero indicate increasing impacts and inner bars below zero indicate decreasing impacts. Countries are grouped by UN georegions.

Areas with fastest increases in CHI nearly always coincide with highest absolute cumulative impacts (Fig. 2, red areas), for example the Black Sea, Eastern Mediterranean Sea, Canadian Eastern Seaboard, southern Atlantic Ocean, and Southern/Western Australia. These regions are at high risk of ecosystem collapse; indeed, some already have (e.g., the Black Sea^18^). Regions with high but decreasing CHI (Fig. 2, maroon areas) were mostly located throughout the northern latitudes of the Atlantic Ocean, including parts of the North Sea and Norwegian Sea. Areas with low and decreasing CHI (Fig. 2, blue areas), in the Central Pacific, Southern Ocean, and parts of the Russian Arctic, may be especially significant for future management focus as they could play key roles as refugia for marine biodiversity. However, in the high seas of equatorial regions and the Southern Ocean the significant decreases are largely due to local relaxation of climate change stressors, ocean temperature in particular (Fig. S9), and do not appear to persist as global climate change continues to accelerate beyond 2013 (Fig. S10). Few areas exist with low and fast increasing CHI (Fig. 3, yellow areas), mostly as isolated patches in equatorial regions.

Given the global patterns, it is not surprising that just over 85% (N = 190) of the 220 coastal countries and territories (hereafter ‘countries’, Fig. 3, Table S7) experienced average increasing rates of CHI within their 3 nm coastal zone, with nearly 10% of these (N = 19) having a very fast average pace of change (>0.1 yr^−1^). Islands in the Caribbean and mid-latitudes of the Indian Ocean experienced the greatest increases (Fig. 3), with Réunion having the fastest pace of increase (0.17 yr^−1^; Table S8).

Climate change stressors are generally increasing rapidly, driving most of the change in CHI at both global and coastal (Fig. 3) scales. At the global scale, increases in the frequency of anomalously high sea surface temperature (SST) events account for about 75% of the observed increase in CHI (Fig. S9, Table S7), and ocean acidification is the next fastest increasing impact, explaining an additional 16% of the increase in CHI (Table S7). At the near-coastal scale (i.e., 3 nm offshore), increases in the frequency of SST events account for nearly 40% of the increase in CHI, and increasing sea level explaining about 41% of the increase (Table S7). In particular, increases in SST explain most of the increase in CHI in the southern hemisphere, especially in high seas regions, and in regions subject to relatively low cumulative impact in 2013, such as the Indian Ocean, Mid-Atlantic and Western Pacific (Fig. S9). Stable temperatures during 2003–2013 underlie the lack of change or decrease in CHI observed throughout much of the Eastern Pacific Ocean (particularly along the west coast of Canada and the U.S.) and Northern Atlantic Ocean. However, these regional patches of pause in ocean warming have already mostly disappeared in recent years^19^ (Fig. S10) – the lack of data for all stressors through 2017 precluded calculating CHI in these later years. Given inertia in the climate system, climate drivers of cumulative impact will likely increase for at least a few decades more, potentially at an accelerating rate, adding further urgency to the need to address climate change and its associated pressures on ocean ecosystems.

Despite the dominant role of climate change pressures, when SST is removed from the analysis, an even larger proportion of the globe (77% vs. 59%) shows a statistically significant increase in cumulative impacts based on the remaining 13 stressors, although the magnitude of change is much lower (Fig. S9). In coastal areas, SST and sea level rise (SLR) explained about 80% of the pace of change in overall CHI trends (Table S7, Fig. 3). However, other stressors play an important role in driving pace of change (Table S7). When SST trends were removed, the cumulative impact of the remaining 13 stressors still increased over time for 92% of countries (Fig. S11; vs. 86% with all stressors), however, the magnitude of the change was smaller in almost all cases (Fig. S12). This consistent significant increase in pressures is alarming, as it points to the fact that stressors other than SST, often functioning at local scales, are significantly increasing across most of the globe, and current management at these scales is doing little to slow the pace of increasing change.

The majority of countries had increasing rates of ocean acidification (99%), shipping (92%), light pollution (90%), and direct human (70%) impacts. Although organic chemical and nutrient pollution from land-based use increased globally, the majority of countries (65% and 56%, respectively) appear to have experienced declines in these impacts. The most impactful forms of demersal fishing (destructive and non-destructive, high-bycatch) declined globally, whereas pelagic high and low bycatch fishing impacts (pelagic and demersal non-destructive) overall increased. Total fishing pressures decreased for many countries, with 53% of countries experiencing declines in 3 or more of the 5 categories of commercial fisheries pressures. Particularly large declines were observed in Singapore, Slovenia, and South Korea. Determining whether management actions (e.g., MPAs, fisheries reform, land use regulations) or other factors, such as declining stocks, drove these declines remains an important area of future research that will require regional and local-scale assessment.

**Figure 4:**
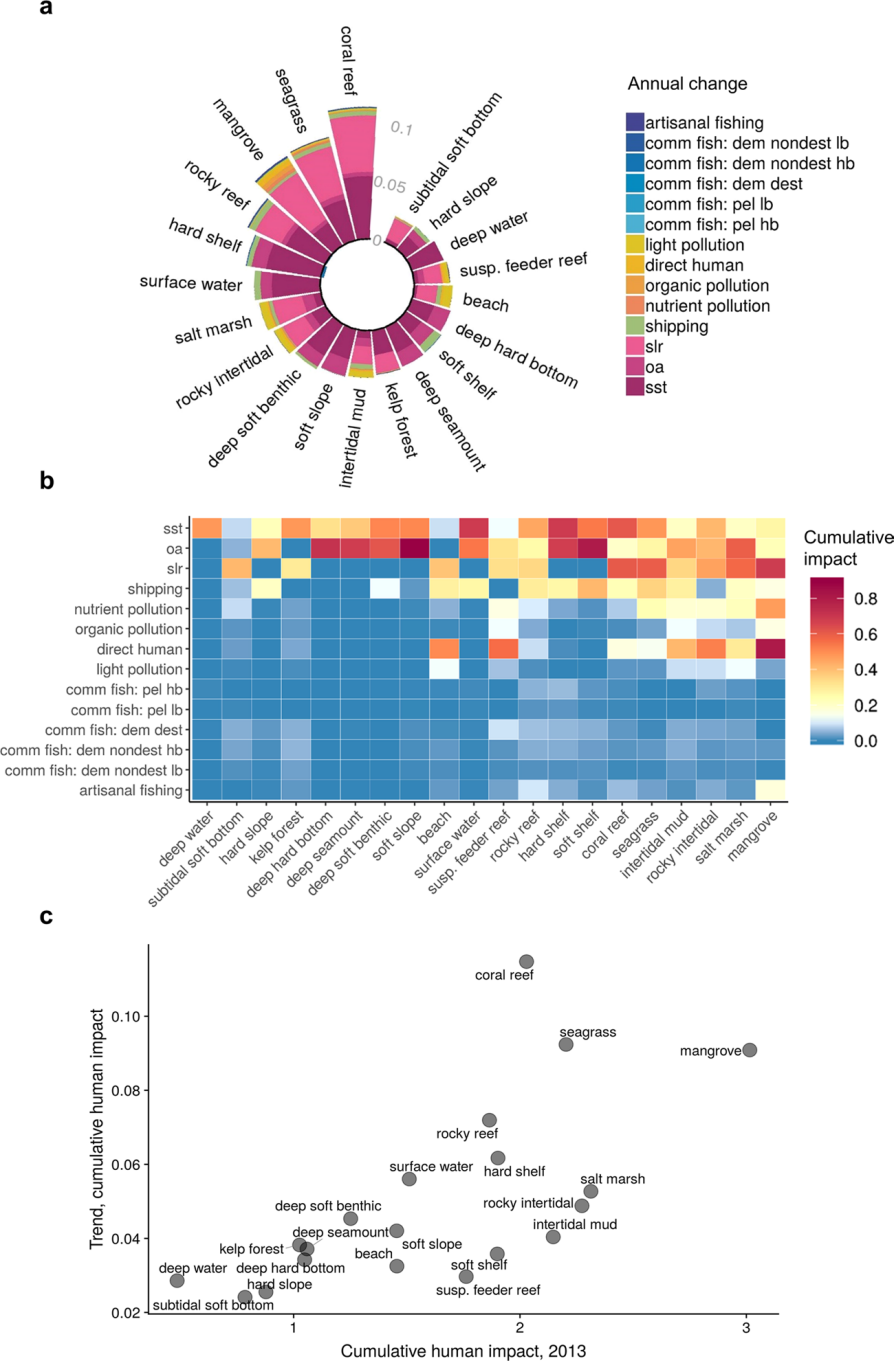
Cumulative human impacts on marine ecosystems. (**a**) Annual change in all 14 impacts comprising the cumulative impacts for each ecosystem, with outer bars above zero indicating increasing impacts and inner bars below zero indicate decreasing impacts, (**b**) cumulative impacts on ecosystems for the current year (2013), and (**c**) relationship between annual trend and current cumulative impacts for each ecosystem.

Coastal ecosystems, in particular coral reefs, seagrasses, and mangroves, experienced the fastest pace of increase in CHI (Fig. 4a,c) as well as the highest average CHI (Fig. 4b,c), highlighting that nearshore ecosystems, often with smaller spatial extent, are the most vulnerable to rapid human impact compared to larger and deeper ecosystem types. Even for coastal ecosystems, climate stressors were dominant drivers of change (Fig. 4b), although land-based pressures and shipping also increased notably for many ecosystems (Fig. 4b).

Mapping the pace of change in cumulative human impacts on the ocean provides a fundamentally novel understanding of current and potential future risks to marine ecosystems and biodiversity. The vast majority of the ocean is experiencing significantly increasing impacts from multiple human stressors (Fig. 1a); much of this area currently remains at relatively modest impact, such that a snapshot view of impact gives a false sense of condition (Fig. 1B). More critically, if current trajectories of change persist, the global cumulative impact of humans on the ocean will be profound and may rapidly push many ocean regions past critical tipping points of sustainability^1,2,4,20^.

Despite these sobering results, messages of hope remain. During this time period, many countries, particularly in Europe, Asia, and parts of Africa, saw notable declines in impacts from commercial fishing, and many countries saw reduced impacts from land-based pollution (Fig. 3). In a few cases, these declines were larger than increases in climate change and other stressors, leading to overall decreases in CHI, and in all cases the declines helped mitigate increases in CHI. Coordinated, comprehensive management that accounts for multiple stressors can leverage decreases in single stressors to accommodate potential increases in others when making strategic development and conservation decisions. Results also highlight that spatial variability in the local manifestation of climate change may offer local refugia that can be targeted for protection and management to ‘buy time’ in efforts to mitigate and adapt to a changing climate^21^. Despite major challenges in reducing greenhouse gas emissions, these results indicate that climate mitigation to meet targets of the Paris Agreement would have major positive impact on the condition of marine ecosystems and would significantly slow or halt increasing trends in CHI in vast areas of the ocean.

Our results are robust to many methodological decisions because our focus was on change in impact over time, as data processing and analytical decisions remain consistent across all years. Furthermore, we have found previously that global patterns and results are robust to model parameters, including stressor vulnerability weights^12,22^. However, for several reasons our results are likely conservative^22^. First, many human activities with known stressors to marine ecosystems could not be included (e.g., deep sea mining, plastic pollution, offshore energy, aquaculture, noise pollution, terrestrial mining, logging, oil spills), primarily because of limited or nonexistent data on the spatial distribution or temporal change in their intensity. Many of these excluded activities have been expanding in geographic extent and intensity over the past decade. Second, our analysis did not include the most recent 5 years of impact because many datasets are not yet available for these years, during which time many are expected or known to have further increased, most notably climate related stressors^23^. Third, multiple interacting stressors often produce synergistic rather than simply additive outcomes^24^, such that increasing intensity of individual stressors within the context of multiple stressors is likely to accelerate CHI faster than we modeled here. Finally, we expect nonlinear relationships to exist between ecosystem condition and the intensity of both individual stressors and CHI^25–27^. These nonlinearities would lead to faster than linear increases in ecosystem impact with increasing stressor intensity that would necessitate lower thresholds for rescaling individual stressors. Very few data or known thresholds currently exist to allow inclusion of these nonlinearities in our assessment.

Previous snapshot views of cumulative human impacts on the ocean^12,13^ have already been widely used to inform where to locate new MPAs^28–30^, new ocean uses within a spatial planning framework^31^, and new conservation or restoration strategies^32^; to assess if existing MPAs are working^33^; and, combined with biodiversity data, to assess species risk to inform Aichi targets and other conservation goals^14^. Understanding the pace of change in human impacts provides a much richer understanding of how, where, and critically how quickly, human activities are affecting marine ecosystems and ultimately the services they provide humanity, thus offering a much more informed baseline to guide strategic conservation actions and assessments.

Looking forward, with human dependencies on land expanding and increasingly leading to conflict, countries around the world are progressively pushing into the ocean, intensifying past uses and adding additional ones – including offshore energy, marine aquaculture, and even human settlements. Such expansions are driven by the need to feed and support a rapidly growing global human population, but come with yet greater impacts on the ocean. This reality requires humanity to face difficult decisions ahead. To help support the global human population and mitigate the impacts we are having on our landscapes, we are shifting our impacts into the sea. How much more change can these ecosystems endure?

## Methods

We calculated change in the intensity of 14 stressors from human-based activities during an 11 year period from 2003 to 2013 at a ~1 km resolution and estimated the impact on the global ocean based on the magnitude of the stressor as well as the vulnerability of 21 marine ecosystems to each stressor. The cumulative impact of all 14 stressors was then calculated for each km^2^ for each year. To determine annual change in stressor and cumulative impacts, we applied a linear regression model to each raster cell and calculate whether the slope is significantly different from 0.

### General model

We calculated stressor and cumulative impact *I*_*c*_ at a ~1 km resolution, based on previously developed methods^12,13^, using the following information:

1. Stressor intensity rasters describing the magnitude of 14 stressors on a scale of 0–1 (1 is highest relative stress).
2. Ecosystem rasters describing the location (1 if present, otherwise NA) of 21 global marine ecosystem types.
3. Vulnerability matrix describing the vulnerability of each ecosystem to each stressor. Vulnerability is a value from 0–4.

Stressor impacts, *I*_*s*_, were calculated for each of the 14 stressors by first multiplying the stressor intensity raster, *D*_*j*_, by each ecosystem raster, *E*_*i*_, and the corresponding vulnerability value, *μ*_*ij*_. The stressor × ecosystem × vulnerability rasters are summed for each stressor and then divided by the number of habitats (*m*) in each raster cell:

$${I}_{s}=\frac{1}{m}\mathop{\sum }\limits_{i=1}^{m}{D}_{j}\times {E}_{i}\times {\mu }_{ij}$$

Cumulative impact, *I*_*c*_, is calculated by summing the 14 stressor impact rasters:

$${I}_{c}=\mathop{\sum }\limits_{i=1}^{m}{I}_{s}$$

### Data representation

All data are represented at ~1 km resolution with a WGS84 Mollweide projection. Nearly all source data used to derive the stressor layers had native coarser resolution (Table S2), and were therefore resampled/reprojected using nearest neighbor estimates of cell values. Using the nearest neighbor approach preserves the values of the original data, and assumes the coarse-scale value is evenly distributed across all 1 km cells within that region. The coarser scale pattern is essentially recreated and finer resolution information is preserved where and when it is appropriate.

We used the WGS84 Mollweide projection because it preserves area so data towards the poles are not visually over-represented.

### Stressors

We include stressors from 4 primary categories:

- Fishing: commercial demersal destructive, commercial demersal nondestructive high bycatch, commercial demersal nondestructive low bycatch, pelagic high bycatch, pelagic low bycatch, artisanal
- Climate change: sea surface temperature, ocean acidification, sea level rise
- Ocean: shipping
- Land-based: nutrient pollution, organic chemical pollution, direct human, light

Stressors included in our analyses are listed in Tables S1 and S2 and described in detail within the Supplementary Information.

Given our focus of describing *trends* in human impact on marine ecosystems, global datasets reporting results at regular intervals were critical. Given this constraint, we were unable to include some stressors from previous impact analyses^12,13^ because they did not include enough information for us to estimate annual change from 2003 to 2013. These excluded stressors include invasive species, ocean pollution, UV intensity, and benthic structures.

Other anthropogenic drivers we considered, but could not be included due to incomplete spatial or temporal coverage, were: hypoxic zones, coastal engineering (piers, rock walls, etc.), non-cargo shipping (ferries, cruise ships, etc), aquaculture, disease, changes in sedimentation and freshwater input, and tourism.

Given discrepancies in how different data layers define the global coastline, we resolved differences by extending all stressor rasters to a common coastline. In some cases the gaps occurred because the monitoring that produced the stressor data missed some regions; however, it was most often due to converting a coarser resolution raster to a finer resolution raster, resulting in zig-zags of missing data along the coast. This made gapfilling necessary. Details for how spatial gapfilling was done for each stressor are provided in the Supplementary Information.

Stressors are rescaled to have values between 0–1. Rescaling allows for direct comparison among drivers with dramatically different units of measurement. With the exception of ocean acidification, we rescaled the data by dividing by the 99.99th quantile across all global raster cells and years (values are capped to a maximum value of 1). We used all years of data to rescale the data to ensure comparability across time periods. The 99.99th quantile was used to minimize the influence of outliers. This approach assumes a linear relationship between the magnitude of the stressor and the impact on the ecosystem. This assumption ignores thresholds that likely exist but are known for very few stressors. For ocean acidification we used known information about biological thresholds to rescale the data.

For many stressors, the distribution of values was highly skewed such that rescaling relative to the highest values resulted in intermediate values of the stressor that were underestimated. In these cases, we log transformed stressor values prior to rescaling. We indicate when data were transformed in descriptions of each stressor in the Supplementary Information.

## Supplementary information

Supplementary Information
