# Supplementary material for: Recent pace of change in human impact on the world’s ocean
Source: Sci Rep. 2019 Aug 12;9:11609. doi: 10.1038/s41598-019-47201-9 (PMC6691109; doi:10.1038/s41598-019-47201-9)
Supplement: Supplementary file 1 — Supplementary Information [file 41598_2019_47201_MOESM1_ESM.docx]

# Supplementary Materials

Recent pace of change in human impact on the world’s ocean

Benjamin S. Halpern^*^, Melanie Frazier, Jamie Afflerbach, Julia S. Lowndes, Fiorenza Micheli, Casey O’Hara, Courtney Scarborough, Kimberly A. Selkoe

*Corresponding author. Email: halpern@nceas.ucsb.edu

**This file includes:**

Materials and Methods

Figs. S1 to S14

Tables S1 to S7

References

# Supplementary Materials and Methods

We describe change in the intensity of human-based activities on the global ocean during an 11-year period from 2003 to 2013 at a ~1km resolution. Specifically, we estimated the impact of 14 stressors based on the magnitude of the stressor as well as the vulnerability of 21 marine ecosystems to the stressor. The cumulative impact, which describes the impact of all stressors, was estimated by summing all 14 stressor impacts for each year. To determine annual change in stressor and cumulative impacts, we applied a linear regression model to each raster cell.

For more information about methods see Halpern et al. [2008](http://science.sciencemag.org/content/319/5865/948) and [2015](https://www.nature.com/articles/ncomms8615).

**General model**

We calculate stressor and cumulative impact at ~1km resolution, using the following information (Fig S1):

1. Stressor intensity rasters describing the magnitude of 14 stressors on a scale of 0-1 (1 is highest relative stress).
2. Ecosystem rasters describing the location (1 if present, otherwise NA) of 21 global marine ecosystems.
3. Vulnerability matrix describing the vulnerability of each ecosystem to each stressor. Vulnerability is a value from 0-4.

***Fig S1. Methods of estimating impact***

*We estimated the impact of 14 stressors based on stressor intensity and the vulnerability of 21 marine ecosystems to the stressor. Total cumulative impacts are estimated by summing all stressor impacts.*


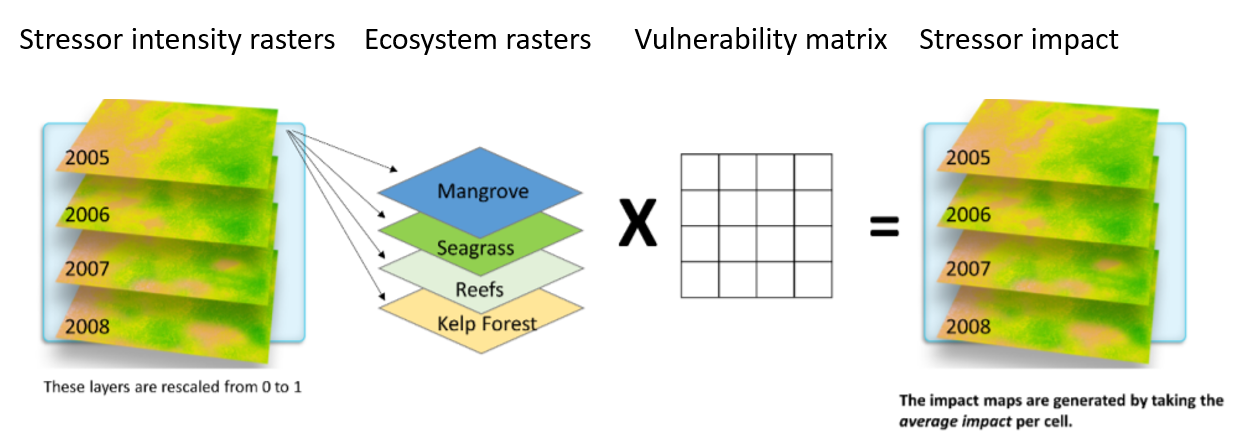


Impact rasters, $I_{s}$, were calculated for each of the 14 stressors by first multiplying a stressor intensity raster, $D_{j}$, by each ecosystem raster, $E_{i}$, and the corresponding vulnerability value, $\mu_{ij}$. The $n$=21 stressor x ecosystem x vulnerability raster combinations are summed and divided by the number of habitats, $m$, in each raster cell:

$$I_{s}=\frac{1}{m}\sum_{i=1}^{n} D_{j}\times E_{i}\times\mu_{ij}$$

The cumulative impact raster, $I_{c}$, is calculated by summing the 14 stressor impact rasters:

$$I_{c}=\sum_{i=1}^{n} I_{s}$$

**Data representation**

All data are represented at ~1 km resolution with a WGS84 Mollweide projection. Nearly all of the source data used to derive the stressor layers had native coarser resolution (Table S2), and were therefore resampled/reprojected using nearest neighbor estimates of cell values. Using the nearest neighbor approach preserves the values of the original data, and assumes the coarse-scale value is evenly distributed across all 1 km cells within that region. The coarser scale pattern is essentially recreated and finer resolution information is preserved where and when it is appropriate.

We use the WGS84 Mollweide projection because it preserves area so data towards the poles are not visually over-represented.

**Stressors**

We include stressors from 4 primary categories:

- Fishing: commercial demersal destructive, commercial demersal nondestructive high bycatch, commercial demersal nondestructive low bycatch, pelagic high bycatch, pelagic low bycatch, artisanal
- Climate change: sea surface temperature, ocean acidification, sea level rise
- Ocean: shipping
- Land-based: nutrient pollution, organic chemical pollution, direct human, light

Stressors are described in Tables S1 and S2 and methods are provided below.

Given our focus of describing trends in human impact on marine ecosystems, global datasets reporting results at regular intervals were critical. Given this constraint, we were unable to include some stressors from previous impact analyses (Halpern et al. 2008, 2015) because they did not include enough information to estimate annual change from 2003 to 2013. These include pressures requiring port traffic data (invasive species and ocean pollution), UV intensity, and benthic structures.

Other anthropogenic drivers we considered, but could not include due to incomplete spatial or temporal coverage, were: hypoxic zones, coastal engineering (piers, rock walls, etc.), non-cargo shipping (ferries, cruise ships, etc), aquaculture, disease, changes in sedimentation and freshwater input, and tourism.

Many of the habitats are only found near the coast, and to avoid biasing the results the stressor raster data must extend all the way to the coastline (Frazier et al. 2016). In most cases, there were gaps in the data along the coastline. In some cases, this occurred because monitoring missed some regions; however, it was most often due to converting a coarser resolution raster to a finer resolution raster, resulting in zig-zags of missing data along the coast. This made gapfilling necessary. We describe the methods used to gapfill each stressor.

Stressors are rescaled to have values between 0-1. Rescaling allows for direct comparison among drivers with dramatically different units of measurement. With the exception of ocean acidification, we rescaled the data by dividing by the 99.99th quantile across all global raster cells and years (values are capped to a maximum value of 1). We used all years of data to rescale the data to ensure comparability across time periods. The 99.99th quantile was used to minimize the influence of outliers. This approach assumes a linear relationship between the magnitude of the stressor and the impact on the ecosystem. This assumption ignores thresholds that likely exist but are known for very few stressors. For the ocean acidification stressor we were able to apply information about biological thresholds to rescale the data.

For many stressors, the distribution of values was highly skewed such that the highest, most extreme, values dictated the rescaling value. The result was that magnitude of intermediate values of the stressor were underestimated after rescaling. In these cases, we log transformed stressor values prior to rescaling. We indicate when data were transformed in descriptions of each stressor.

**Table S1: Methods used to generate stressor data**

| **stressor** | **preparing data** | **gapfilling** | **rescaling (0 to 1)** | **transformations** |
| --- | --- | --- | --- | --- |
| sea surface temperature | Number of extreme SST weeks during a five-year period subtracted from the number of extreme SST weeks during a baseline 5 year period (1985-1989). An extreme week is defined as having a SST anomaly (weekly SST - weekly climatological SST) exceeding 1 SD of the anomalies calculated across all years (1982-2017) for that week. | Coastal cells without data estimated using mean of surrounding cells, gapfilling performed on total annual extreme events | Raster value corresponding 99.99th quantile across all years | NA |
| ocean acidification | Monthly aragonite saturation values averaged to obtain annual estimates. | Coastal cells without data estimated using inverse distance weighting of rescaled data | Cell values ≤ 1 are given a pressure score of 1; for all other cells, rescaling is based on the change in saturation relative to the baseline period (average from 1880-1889) | NA |
| sea level rise | Monthly anomalies averaged to obtain annual mean sea level anomaly. 5 year mean of annual data used to smooth large yearly variation. | Estimated missing coastal data using inverse distance weighting of 5 year mean anomaly data | Raster value corresponding 99.99th quantile across all years | NA |
| fishing: artisanal | Total tonnes of catch from nonindustrial fisheries calculated for each year. Catch divided by corresponding year’s Net Primary Productivity (monthly data averaged for yearly NPP estimate) to standardize by region’s productivity. | Fisheries catch and NPP gapfilled using average of nearest neighbors | Raster value corresponding 99.99th quantile across all years | NPP log10, catch not transformed |
| fishing: demersal destructive | Total tonnes of catch for industrial demersal fishing using gear types causing habitat destruction calculated for each year. Catch data divided by corresponding year’s Net Primary Productivity (monthly data averaged for yearly NPP estimate) to standardize by region’s productivity. | Fisheries and NPP gapfilled using average of nearest neighbors | Raster value corresponding 99.99th quantile across all years | NPP log10, catch not transformed |
| fishing: demersal nondestructive high bycatch | Total tonnes of catch for industrial demersal fishing using high bycatch practices calculated for each year. Catch data divided by corresponding year’s Net Primary Productivity (monthly data averaged for yearly NPP estimate) to standardize by region’s productivity. | Fisheries and NPP gapfilled using average of nearest neighbors | Raster value corresponding 99.99th quantile across all years | NPP log10, catch not transformed |
| fishing: demersal nondestructive low bycatch | Total tonnes of catch for industrial demersal fishing using low bycatch practices calculated for each year. Catch data divided by corresponding year’s Net Primary Productivity (monthly data averaged for yearly NPP estimate) to standardize by the region’s productivity. | Fisheries and NPP gapfilled using average of neighbors | Raster value corresponding 99.99th quantile across all years | NPP log10, catch not transformed |
| fishing: pelagic high bycatch | Total tonnes of catch for industrial pelagic fishing using high bycatch practices calculated for each year. Catch data divided by corresponding year’s Net Primary Productivity (monthly data averaged for yearly NPP estimate) to standardize by region’s productivity. | Fisheries and NPP gapfilled using average of nearest neighbors | Raster value corresponding 99.99th quantile across all years | NPP log10, catch not transformed |
| fishing: pelagic low bycatch | Total tonnes of catch for industrial pelagic fishing using low bycatch practices calculated for each year. Catch data divided by corresponding year’s Net Primary Productivity (monthly data averaged for yearly NPP estimate) to standardize by region’s productivity. | Fisheries and NPP gapfilled using average of neighbors | Raster value corresponding 99.99th quantile across all years | NPP log10, catch not transformed |
| nutrient pollution (runoff) | Intensity of pollution from modeled plumes of land-based fertilizer pollution (Halpern et al. 2008), based on country level fertilizer use (UN 2016), land cover data (Friedle et al. 2010), and elevation data (USGS 2004) | NA | Raster value corresponding to 99.99th quantile across all years | ln(x + 1) |
| organic chemical pollution (runoff) | Intensity of pollution from modeled plumes of land-based pesticide pollution (Halpern et al. 2008), based on country level pesticide use (UN 2016), land cover data (Friedle et al. 2010), and elevation data (USGS 2004) | NA | Raster value corresponding to 99.99th quantile across all years | ln(x + 1) |
| direct human | Density (people per km^2^) converted to population (people per raster cell). Intervening years (i.e., years outside of 2000, 2005, 2010, 2015, 2020) interpolated using a linear model. For each raster cell, coastal human population summed for 10 km radius. Data cropped to include only cells 1km from the coast. | NA | Raster value corresponding to average 99.99th quantile across all years | ln(x + 1) |
| light | Non-calibrated radiance values from satellite data calibrated across year/satellite following methods of Elvidge et al. 2009. | The dataset does not include the north and south pole regions, and these were estimated as zero | Raster value corresponding 99.99th quantile across all years (excluding 0 values) | NA |
| shipping | Tournadre (2018) data used to create yearly rasters describing annual proportional change in shipping relative to 2011. Multiplied yearly proportional change raster with high resolution shipping raster (Halpern et al. 2015 and Wallbridge 2013) to estimate shipping traffic over time. | Outer border of shipping data was gapfilled using mean of nearest neighbors; NA values for yearly shipping density were estimated as the total global change in shipping relative to 2011 | 99.99th quantile determined for each year (1992-2016) and the average of these values used to rescale data | ln(x + 1) |

**Table S2: Data sources for stressor data**

| **Stressor** | **Source** | **short description** | **spatial resolution** | **temporal resolution** |
| --- | --- | --- | --- | --- |
| Sea surface temperature | Selig et al. 2010; Saha et al. 2018 | weekly SST anomalies (weekly SST minus weekly climatological SST) | approximately 4.6 km at the equator | weekly, 1982-2017 |
| Ocean acidification | Feely et al. 2009 | aragonite saturation state | 1° | monthly, 1880-2020 |
| Sea level rise | Aviso 2016 | mean sea level anomaly (meters, current monthly sea level minus average monthly sea level from 1993 - 2016) | 0.25° | monthly, 1993-2016 |
| Fishing: artisanal | Watson 2018 (catch data); Behrenfeld and Falkowski 1997 (net primary productivity) | catch: nonindustrial tonnes of catch; NPP: C/m2/day | catch: 0.5°; NPP: 0.83° | catch: yearly, 1869-2015; NPP: monthly, 2003-2015 |
| Fishing: commercial demersal destructive | Watson 2018 (catch data); Behrenfeld and Falkowski 1997 (net primary productivity) | catch: industrial tonnes of catch per taxa and gear; NPP: C/m2/day | catch: 0.5°; NPP: 0.83° | catch: yearly, 1869-2015; NPP: monthly, 2003-2015 |
| Fishing: commercial demersal nondestructive high bycatch | Watson 2018 (catch data); Behrenfeld and Falkowski 1997 (net primary productivity) | catch: industrial tonnes of catch per taxa and gear; NPP: C/m2/day | catch: 0.5°; NPP: 0.83° | catch: yearly, 1869-2015; NPP: monthly, 2003-2015 |
| Fishing: commercial demersal nondestructive low bycatch | Watson 2018 (catch data); Behrenfeld and Falkowski 1997 (net primary productivity) | catch: industrial tonnes of catch per taxa and gear; NPP: C/m2/day | catch: 0.5°; NPP: 0.83° | catch: yearly, 1869-2015; NPP: monthly, 2003-2015 |
| Fishing: commercial pelagic high bycatch | Watson 2018 (catch data); Behrenfeld and Falkowski 1997 (net primary productivity) | catch: industrial tonnes of catch per taxa and gear; NPP: C/m2/day | catch: 0.5°; NPP: 0.83° | catch: yearly, 1869-2015; NPP: monthly, 2003-2015 |
| Fishing: commercial pelagic low bycatch | Watson 2018 (catch data); Behrenfeld and Falkowski 1997 (net primary productivity) | catch: industrial tonnes of catch per taxa and gear; NPP: C/m2/day | catch: 0.5°; NPP: 0.83° | catch: yearly, 1869-2015; NPP: monthly, 2003-2015 |
| Nutrient pollution (runoff) | Halpern et al. 2008 | Relative intensity of pollution from modeled plumes of land-based fertilizer pollution | ~ 1km | yearly, 2002-2016 |
| Organic chemical pollution (runoff) | Halpern et al. 2008 | Relative intensity of pollution from modeled plumes of land-based organic chemical pollution | ~ 1km | yearly, 2002-2016 |
| Direct human | CIESIN 2017 | Human density, number of persons per square kilometer | 30 arc-second | 2005, 2010, 2015, and 2020 |
| Light | Elvidge et al. 2009; image and data processing by NOAA’s National Geophysical Data Center | non-calibrated radiance values from 0-63 | 30 arc-second spanning -180 to 180 degrees longitude and -65 to 75 degrees latitude | yearly, 1992-2013 |
| Shipping | Halpern et al. 2015 and Wallbridge 2013 (high resolution shipping data); Tournadre 2018 (low resolution annual shipping data) | Tournadre, ships per cell, such that sum of cells represents all ships at sea in given year; Halpern et al. & Walbridge, vessel track density | Halpern: 0.1°; Tournadre: 1x2° | Halpern: combined years (1991-2011, but mostly 2011); Tournadre: yearly, 1992-2016 |

***Stressor layer descriptions***

*Fishing*

Commercial fishing (5 stressors)

There are 5 commercial fishing stressors that have different impacts on marine ecosystems:

- demersal destructive
- demersal non-destructive high bycatch
- demersal non-destructive low bycatch
- pelagic high bycatch
- pelagic low bycatch

The stressors were calculated using annual wild caught industrial fisheries catch (tonnes) by taxa and gear type at 0.5° resolution (Watson 2018, Global Fisheries Landings V3.0; Watson and Tidd 2018). For each stressor we summed the relevant industrial tonnes of catch for each raster cell based on gear type and, in some cases, taxonomic information (Table S3).

**Table S3. Gear types provided by Watson (2018) used to assign catch to each commercial fisheries pressure**


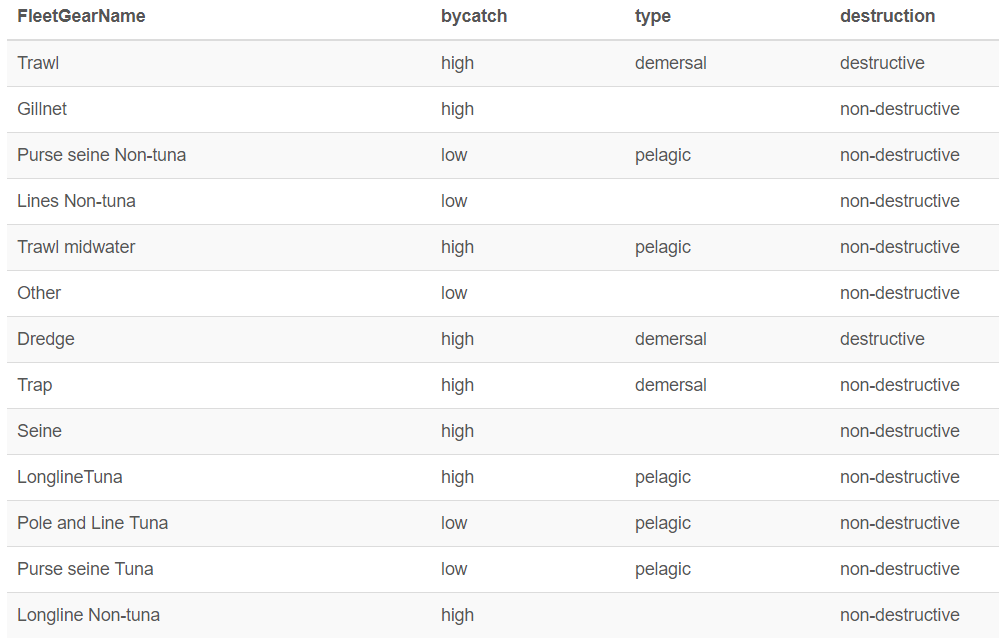


For catch records we were unable to confidently assign to the demersal or pelagic category based on gear type, we determined the taxa’s habitat using the “functional group” from the Sea Around Us taxon database (Pauly & Zeller 2015, seaaroundus.org).

Catch (landings) data are only a proxy for the potential impact of fishing on marine ecosystems because the effort to land the catch determines impact. For example, an area with low fish densities would have to be trawled many more times to harvest an equal catch to an area with higher fish densities. To attempt to control for this issue, we divide catch by the corresponding year’s Net Primary Productivity (NPP, Behrenfeld and Falkowski 1997). Daily data are provided for NPP. These values are log10 transformed and daily values are averaged to obtain a yearly estimate.

There were some coastal raster cells without data. For these cells, we estimated the total catch within each stressor using the mean of surrounding cells (Fig S2).

***Fig S2. Gapfilled cells for fisheries data***

*Red areas indicate cells estimated using surrounding data.*
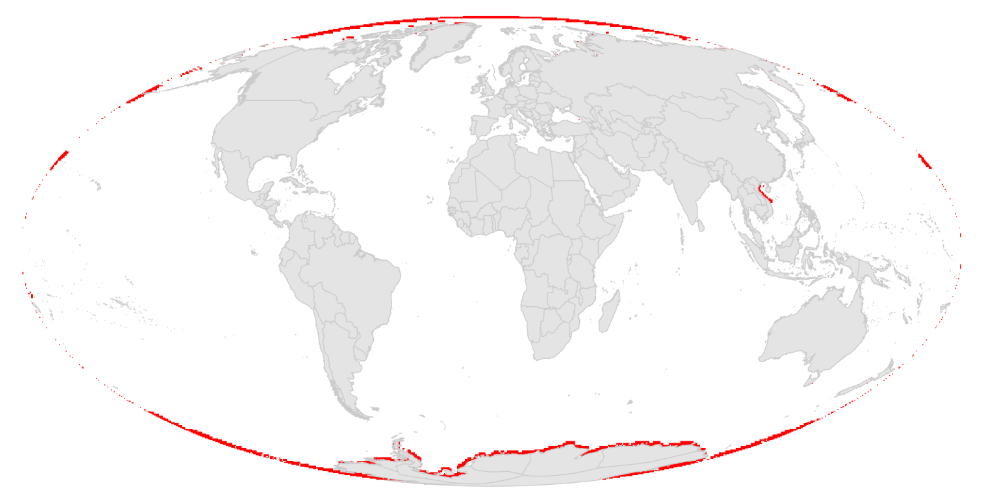


Artisanal fishing

The artisanal fishing stressor was calculated using annual wild caught nonindustrial fisheries catch (tonnes) at 0.5° resolution (Watson 2018). We summed nonindustrial tonnes catch (Watson 2018) for each raster cell, and otherwise, followed the methods used to estimate the commercial fishing stressors.

Gapfilled cells are the same as those from the commercial fishing stressors, described above.

*Climate change*

Sea surface temperature

The sea surface temperature (SST) stressor describes the increased frequency of extreme temperature events relative to a historical baseline period. We used weekly SST anomaly data from version 6 of the Coral Reef Temperature Anomaly Database (CoRTAD). These data are a product of the NOAA National Oceanographic Data Center, produced using Advanced Very High Resolution Radiometer (AVHRR) Pathfinder SST data (<https://data.nodc.noaa.gov/cgi-bin/iso?id=gov.noaa.nodc:NCEI-CoRTADv6>). SST anomalies are defined as the weekly SST minus weekly climatological SST, as calculated across all years of data (1982-2017).

The number of weeks, during a five year period, with extremely warm SST was subtracted from the number of extreme SST weeks during a five year baseline period (1985-1989). An extreme SST event was defined as a week when the SST anomaly exceeded the standard deviation of SST anomalies for that location (i.e., grid cell) and week of the year. Extreme events included only high (i.e., warming) SST events.

We use 5-year periods (e.g., 2013 data includes extreme events from 2009-2013) to smooth yearly variation that is not due to long-term climate change.

There were some coastal raster cells without data. For these cells, we estimated the total annual extreme events using the mean of surrounding cells (Fig S3).

***Fig S3. Gapfilled cells for sea surface temperature data***

*Red areas indicate cells estimated using surrounding data.*


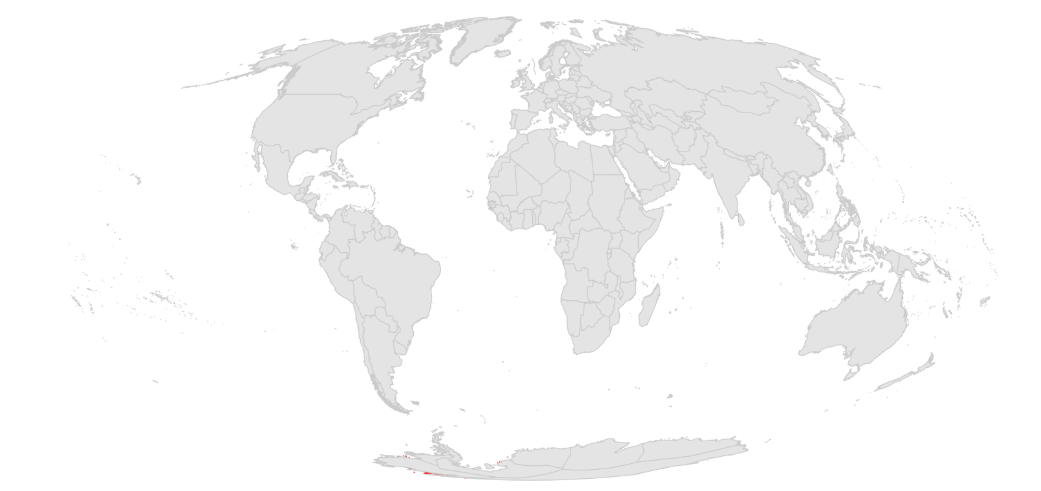


Ocean acidification

The ocean acidification stressor describes the magnitude of decreasing aragonite saturation state (*Ωarag*) due to increasing atmospheric CO_2_ levels from human influences. Monthly data are provided at 1° resolution (Feely et al. 2009). Monthly data are averaged to obtain yearly estimates of aragonite saturation state.

This stressor is unique in that the data are rescaled to values between 0 and 1 using a biological threshold rather than the 99.99th quantile across raster cells and year. When values are ≤ 1, the threshold at which seawater becomes undersaturated, the pressure is 1 (the highest pressure). For values >1, rescaling is based on the change in saturation relative to a baseline period (average from 1880-1889), using the following formula:

$$Stress= \frac{(\Omega_{baseline}-\Omega_{year})}{(\Omega_{baseline}-1)}$$

The current value is subtracted from the baseline so a reduction in *Ω* becomes a positive pressure value. This value is then divided by the baseline minus one so a decrease in *Ω* while the current state is relatively high results in lower pressure than the same decrease when the current state is near 1. Increases in aragonite saturation state result in stressor = 0.

There were some coastal raster cells without data. For these cells, we estimated the annual stressor values using an inverse distance weighting method (Fig S4).

***Fig S4. Gapfilled cells for ocean acidification***

*Red areas indicate cells estimated using surrounding data.*


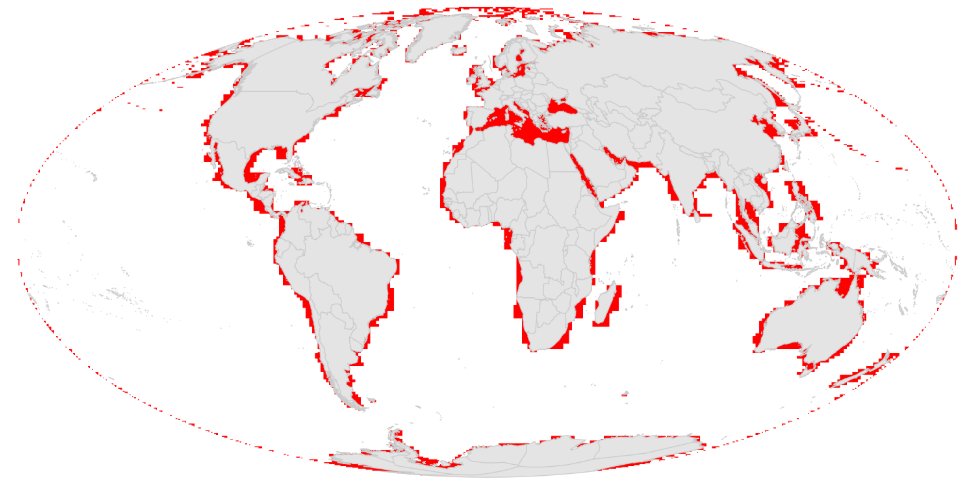


Sea level rise

The sea level rise stressor describes the magnitude of increasing sea level using altimetry data (<http://www.aviso.altimetry.fr/en/data/products/sea-surface-height-products/global/msla-mean-climatology.html>) at a 0.25x0.25° resolution. Data describe monthly sea level anomalies (m), calculated by subtracting monthly sea level from the average sea level calculated from 1993-2016 for each month. For each year, monthly anomalies were averaged to obtain the average annual sea level rise anomaly.

We ultimately used 5 years of anomaly data to calculate each year of stressor data, such that the 2013 stressor layer includes 2009-2013 data, due to large yearly variation in SLR.

All negative values were set to zero (i.e., no negative pressure), such that only positive sea level rise values are considered.

There were some cells without data. For these cells, we estimated missing 5 year average sea level rise anomalies using an inverse distance weighting method (Fig S5).

***Fig S5. Gapfilled cells for sea level rise***

*Red areas indicate cells estimated using surrounding data.*


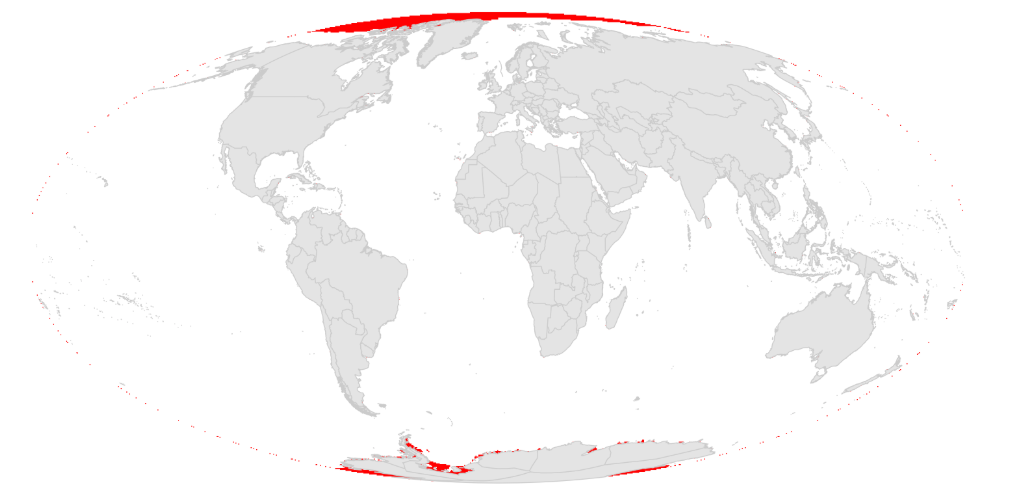


*Ocean-based*

Shipping

This pressure describes intensity of global shipping traffic. High quality, freely available, annual shipping data are currently unavailable. Consequently, we combined two sources of data to describe spatial and temporal shipping patterns.

The first data source was a high resolution (0.1°) map of global vessel traffic based on data from the Volunteer Observing System (VOS) and Automatic Identification System (AIS) (data from Halpern et al. 2015; Wallbridge 2013). These data combine several years of shipping data but are mostly a static description of shipping during 2011. The outermost border of cells was gapfilled using mean of nearest neighbors.

We used a second data source to estimate change over time. Tournadre (2018) provides annual ship densities at 1 x 2° resolution. These data capture temporal shipping patterns, but the spatial and temporal resolution is very low and stochastic due to being based on ship density data. Given this, we used the Tournadre data to create yearly rasters describing the proportional change in shipping patterns relative to a baseline year of 2011, which corresponded to the main year of data used to generate the static shipping raster. To control for temporal stochasticity of these data we averaged 3 year time periods, such that the 2013 proportional change raster is based on 2011-2013 shipping data. The 2011 baseline year was the average of 5 years of data (2009-2013). Averaging multiple years of data didn’t fully compensate for stochasticity, as evidenced by cells with no shipping data when it was obvious there would have been shipping traffic with higher resolution data. In these cases, we estimated the missing cell values with the total global change in shipping relative to 2011 (i.e., total ships in given year/total ships 2011).

To obtain high resolution annual shipping data, we then multiplied the high resolution static shipping raster (Halpern 2015) by each yearly proportional change raster to estimate change over time.

Data were ln(x+1) transformed due to extreme skew.

*Land-based*

Nutrient pollution

The nutrient pollution stressor describes relative intensity of nutrient pollution due to runoff from land-based applications. These data represent modeled nutrient plumes resulting from land-based nitrogen application at ~1km resolution. A brief overview of the methods are provided below, but see Halpern et al. (2008, 2015) for more information.

The plume model uses country-level average annual fertilizer use (as a proxy for nutrient pollution) from FAO (<http://faostat3.fao.org/faostat-gateway/go/to/browse/R/*/E>). Missing values were estimated using a linear regression model between fertilizer and pesticides when possible, and otherwise with agricultural GDP. Data were summed across all fertilizer compounds and reported in metric tons. Upon inspection the data included multiple 0 values that are most likely data gaps in the time-series, so they were treated as such and replaced with NA. Uninhabited countries were assumed to have no fertilizer use and thus excluded.

Country-level pollution values were then dasymetrically distributed over a country’s landscape using global landcover data, derived from the Moderate Resolution imaging Spectroradiometer (MODIS) instrument at ~500m resolution (Channan et al. 2014, Friedl et al. 2010).

The gridded data of pollution was aggregated by summing the raster cell values located within watershed boundaries. Boundaries for ~140,000 global basins were developed with an automated flow-accumulation process (Jenson and Domingue 1988) on the basis of 30 arc-second (nominally 1km^2^) SRTM30 Digital Elevation Model (DEM) data (USGS 2004). The dispersion of the pollution into the ocean was then modeled from each basin’s pourpoint (i.e., location where freshwater enters the ocean). Dispersion into coastal waters from each pour point was modeled with a cost-path surface on the basis of a decay function that assigns a fixed amount of the driver (in our case, 0.5% of the value in the previous cell) in the initial cell and then evenly distributes the remaining amount of driver in all adjacent and ‘unvisited’ cells, repeated until a minimum threshold (0.05% of global maximum) is reached. This approach to modeling river plumes is diffusive and so allows drivers to wrap around headlands and islands, but does not account for nearshore advection that acts to push drivers in particular directions. Because nearshore circulation patterns are known for only a few small regions of the world, we conservatively used this diffusive model.

The ocean pollution raster data are presented at a ~1km Mollweide WGS84 projection.

Data were ln(x+1) transformed.

Organic chemical pollution

The organic chemical pollution stressor describes the amount of organic chemical pollution from land-based applications and run-off. We use FAO data describing country-level pesticide use as a proxy measure for organic pollution. Otherwise, the methods are the same as those for nutrient pollution.

Direct human

This stressor estimates the magnitude of direct human interactions on coastal and near-coastal habitats, such as trampling. We used human density data at 30 arc-second resolution (UN WPP-Adjusted Population Density, v4.0, CIESIN 2017) for years 2005, 2010, 2015, and 2020. Density data were resampled to ~1 km raster resolution and transformed to a Mollweide coordinate reference system using nearest neighbor estimates of cell values. Intervening years of data (i.e., those outside of 2005, 2010, 2015, and 2020) were linearly interpolated.

Population density was converted to population (people per cell) based on raster cell size. We summed the coastal human population, defined as the number of people within a moving circular window around an arbitrary focal coastal cell of radius 10 km. Data were cropped to include only cells 1 km from the coast because this stressor primarily affects intertidal and very nearshore ecosystems.

Values were ln(x+1) transformed.

Light

The light stressor describes the amount of light pollution in marine environments. We used annual DMSP stable night lights satellite data (Elvidge et al. 2009; [https://ngdc.noaa.gov/eog/data/web_data/v4composites/%s.v4.tar](https://ngdc.noaa.gov/eog/data/web_data/v4composites/%25s.v4.tar), image and data processing by NOAA’s National Geophysical Data Center; DMSP satellite data collected by US Air Force Weather Agency) with resolution of 30 arc-seconds spanning -65 to 75 degrees latitude and -180 to 180 degrees longitude. The original data contained non-calibrated radiance values, ranging from 0-63, with different satellites used to collect the data across years.

For each year, the original raster values of 0-63 ($DNo$) were calibrated to produce adjusted, $DNa$, values, using the following formula:

$$DNa=C0+C1*DNo+C2*{DNo}^{2}$$

C0, C1 and C1 coefficients used to calibrate the data were estimated using data only from the Sicily region which was selected because it is not light saturated and is thought to have remained fairly constant over the years. Coefficients for each year were estimated using a 2nd degree polynomial regression model, with the F121999 satellite as a predictor variable, with the model coded in R as:

DNo_year_ ~ 0 + poly(DNo_F121999_, 2, raw=TRUE)

The intercept was forced through the origin, so cells with zero light were estimated as zero values.

We followed Elvidge et al. (2009) and used the F121999 satellite as the reference based on their assessment, that:


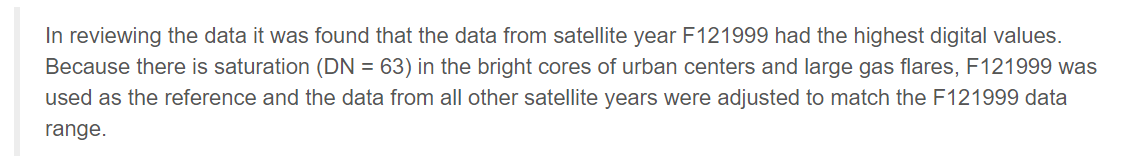


This approach assumes the reference area of Sicily has had little change in light output during this period. This technique, while not perfect, does allow for better comparison between different satellites and time periods.

There is the possibility of missing cells due to cloud cover. These have a value of 255 in the data. For the most part, these cells do not appear in the ocean. In these rare cases, missing cell values were replaced with zero. These data do not fully cover the north and southpole regions, which were estimated as zero values.

**Marine ecosystems**

20 of the 21 marine ecosystems used to estimate impacts remain unchanged from previous impact assessments (Halpern et al. 2008, 2015; Table S4). This year we added a sea ice edge ecosystem, which currently functions as a placeholder because the pressures included in the assessment have limited influence on seaice habitat, which is known to be rapidly changing. We currently do not include UV or air temperature data. We do include SST, but these data are inaccurate in the presence of sea ice. For this reason, we do not include this habitat in our summaries.

The marine ecosystem data is represented as ~1 km Mollweide rasters with ecosystem presence indicated by values of 1 (otherwise values are NA). However, because sea ice is so temporally variable, values range from 0-1 based on the proportion of the year that sea ice edge is present.

**Table S4. Marine ecosystems**

|  | **Habitat** | **year assessed** |
| --- | --- | --- |
|  | sea ice | 2018 |
| **Intertidal** | rocky intertidal | modeled |
|  | beach | modeled |
|  | mud flats | modeled |
|  | salt marsh | modeled |
|  | mangroves | 2006 |
| **coastal**  **(<60 me depth)** | seagrass beds | 2006 |
|  | shellfish reefs | modeled |
|  | coral reefs | 2006 |
|  | rocky reefs | 2005 |
|  | kelp forests | modeled |
|  | shallow soft bottom | 2005 |
|  | shallow pelagic | 2013 |
| **offshore (≥60m depth)** | continental shelf: hard bottom (60-200m) | 2005 |
|  | continental shelf: soft bottom (60-200m) | 2005 |
|  | continental slope: hard bottom (200-2000m) | 2005 |
|  | continental slope: soft bottom (200-2000m) | 2005 |
|  | deep benthic: hard bottom (>2000m) | 2005 |
|  | deep benthic: soft bottom (>2000m) | 2005 |
|  | deep pelagic (>60m) | 2013 |
|  | seamounts | 2004 |

Global data for marine ecosystems are largely non-existent and thus a variety of data and methods were used to generate these data. For some ecosystems we used available data, for others we modeled the distribution, and for several intertidal ecosystems we were forced to assume a uniform distribution because no data exist. A brief description of methods is provided, but see Halpern 2008 for more details.

***Sea ice***

We use monthly Sea Ice Concentrations from Nimbus-7 SMMR and DMSP SSM/I-SSMIS Passive Microwave Data collected from 1981 to 2010 (Cavalieri et al. 2016). We identified sea ice edge cells as those with concentrations between 15-80% (based on: <http://seaiceatlas.snap.uaf.edu/glossary>), and assigned them a value of 1 (otherwise 0). Using the classified sea ice edge data, monthly rasters across all years were averaged to determine the proportion of time the raster is classified as sea ice edge habitat. There is a “hole” of missing data at the northern most part of globe due to satellite coverage, this area was classified as 0, indicating solid ice. We estimated shoreline cells without data using the average of nearest neighbors.

***Hard and soft bottom shallow, shelf, slope, and deep (8 ecosystems)***

The Institute of Arctic & Alpine Research at the University of Colorado at Boulder has benthic substrate point samples from scientific and industry grab samples, trawls, and core samples from around the world as part of the dbSEABED project (http://instaar.colorado.edu/~jenkinsc/dbseabed/). At the time of our purchase of these data (June 2005), the database included 544,543 individual measurements of the percent hard or soft sediment type. The spatial distribution of the data was highly heterogeneous, and roughly 65% of the data were 100% soft sediment. Samples with greater than 50% hard substrate were counted as hard; all others were counted as soft. Grid cells were sampled at 2 arc-minutes (~3.7 km, or 13.69 km^2^ per cell, depending on latitude) and each cell was assigned a binary value of hard or soft substrate. This resolution was used to match the ETOPO2 bathymetry data (http://www.ngdc.noaa.gov/mgg/global/relief/ETOPO2/).

We then used kriging to classify unsampled locations as either soft or hard on the basis of the distribution of substrate types in the surrounding area. Kriging is a statistical interpolation technique for spatial data that uses the spatial variogram of nearby data to assign values to empty cells. Given the vast size of this database, it was not possible to calculate a single variogram for the entire planet, and so we developed variograms for six data-rich regions of the world (Alaska, Florida, Mediterranean, Great Barrier Reef, the North Sea, and New England) and evaluated the kriging solutions that resulted from each of these variograms for indications of poor interpolation, most notably large areas modeled from a single datum and parabolic fan shapes around clusters of points. We used the variogram that resulted in the fewest of these indicators (from the Florida region) and that was from evenly-distributed data that lay along an unbroken swath of coastline with uniform orientation (which reduces clashing trends in the variogram) to produce maps of hard and soft bottom benthic habitats in four different depth ranges – shallow (0-60 m), shelf (60-200 m), slope (200 – 2000 m), and deep/abyssal (>2000 m). Bathymetry data came from ETOPO2 (2 min. resolution).

***Coral reefs***

The global coral reef atlas (Spalding et al. 2001), compiled by the World Conservation Monitoring Centre at the UN Environment Programme (UNEP-WCMC), combines a large number of coral reef maps from various sources. Quality of the final product is limited by quality of the input cartographic sources, some of which are rather coarse scale. Only 30% of reefs in the atlas were mapped from source data with a resolution of 1:250,000 or higher. Nonetheless, coral reefs represent one of the best global marine habitat datasets and this data layer has been widely vetted and used in marine conservation and science. The data were provided to us by UNEP-WCMC as presence/absence for each 1 km cell; we then clipped these data to our land-sea mask.

***Sea grass beds***

We used UNEP-WCMC’s global atlas of seagrasses to represent distribution of this habitat (Green and Short 2003). The dataset compiles aerial maps and point records of seagrass occurrence from around the world. Because of the heterogeneous nature of the input data and the large number of point data in the dataset, the spatial accuracy of the seagrass data layer is limited. In addition, many parts of the world have not been comprehensively surveyed for seagrasses, so this dataset underestimates the distribution of seagrass habitat. Data were overlaid on a 1 km grid and cells were assigned presence or absence of seagrass beds and then clipped to the land-sea mask.

***Mangroves***

Mangrove habitats were mapped with the global mangrove atlas produced by UNEP-WCMC (Spalding et al. 1997). Like the other UNEP-WCMC products, this dataset compiles multiple cartographic sources, derived from aerial imagery, remote sensing, on the ground surveys and expert opinion. This dataset is considered more accurate that the seagrass dataset (i.e., more entries are polygons instead of points). Nonetheless, when we intersected the mangrove layer with our global coastline layer to eliminate mangroves that fell on land, a large portion of the habitat was lost, and there is some error in the original mangrove data. Only 22,139 cells containing mangrove remained. Potential errors in processing this data layer will have a minor effect on global results, because of the small number of cells, but this suggests caution in drawing conclusions about global and regional status of mangrove ecosystems. These data were overlaid on a 1 km grid and cells were assigned presence or absence of mangroves.

***Surface water***

Pelagic surface waters were mapped as the top 60 m of the ocean in all areas deeper than 60 m total depth. Actual depth of this layer can vary from a few meters in turbid water up to 200 m in highly oligotrophic waters (i.e., most of the open ocean) but is commonly shallower in coastal waters where primary productivity (and therefore turbidity) is greater. We used 60 m to be inclusive. The choice of this depth will only affect the distribution of this ecosystem in coastal waters, and this is also where slight inaccuracies may exist due to variation in the quality of the underlying bathymetric data used to map the 60 m depth contour. The water column over shallow (<60 m) locations was excluded because we assumed that the entire shallow water column acts roughly as a single (benthic-associated) ecosystem.

***Deep water***

Pelagic deep waters were defined as the water column from 60 m depth down to the benthos. Like pelagic surface waters, this habitat layer is well mapped, though slight inaccuracies may have been introduced by errors in the bathymetry along the 60 m depth contour. Surface and deep pelagic water habitats are completely overlapping in the horizontal dimension.

***Seamounts***

Seamount location data came from other research efforts (Kitchingman and Lai 2004) that used ETOPO2 bathymetric data (http://www.ngdc.noaa.gov/mgg/global/relief/ETOPO2/) to model seamount location. This research identified peak seafloor anomalies with D8 flow models (Jenson and Domingue 1988) and then compared the output from two different seamount definitions – peaks with >1000 m rise and peaks with roughly circular or elliptical in shape. The former definition focuses on eliminating small peaks that may not be seamounts, and the latter definition focuses on eliminating peaks along steep ridges (e.g., continental slopes). Overlap of these two models produced 14,287 seamounts globally, estimated to represent roughly 30% of likely seamounts in the world (depending on how one defines seamounts). These data were provided to us as the center of each seamount at 1 km^2^ resolution. To better estimate the spatial coverage of these seamounts, we created a small circular buffer around each point, such that each seamount was represented as five 1 km cells centered on the original points and radiating in the cardinal directions.

***Nearshore ecosystems with assumed distributions***

Several coastal and intertidal ecosystems currently have no global data on their spatial distribution but are important for understanding the cumulative impact humans have on the oceans. We included these ecosystems in our cumulative impact model by assuming that rocky intertidal, beach, intertidal mud, suspension-feeding reefs, and salt marsh ecosystems existed in all cells within 1km of shore, and that kelp forest ecosystems exist within all areas <60m deep within their possible range limits (Steneck et al. 2002). We could not include similar range constraints for salt marshes and suspension-feeding reefs because no published records exist for their global ranges.

**Vulnerability matrix**

The vulnerability matrix translates stressor data into impact data. The vulnerability of each marine ecosystem to each stressor is described with integers ranging from 0 (no impact) to 4 (highest impact; Table S5). These weighting variables are from an expert survey that assessed the vulnerability of each ecosystem to each stressor on the basis of 5 ecological traits (Halpern et al. 2008). The vulnerability matrix has remained unchanged from the 2015 cumulative impact analysis (Halpern et al. 2015).

**Table S5. Vulnerability of each ecosystem to each stressor**

|  | Intertidal | | | | | coastal | | | | | | ice, coastal and offshore | offshore | | | | | | | | |
| --- | --- | --- | --- | --- | --- | --- | --- | --- | --- | --- | --- | --- | --- | --- | --- | --- | --- | --- | --- | --- | --- |
|  | rocky intertidal | intertidal mud | Beach | mangroves | salt marsh | coral reefs | seagrass | kelp forest | rocky reef | shellfish reef | shallow soft bottom |  | continental shelf, soft bottom  (60-200m) | continental shelf, hard bottom  (60-200m) | continental slope, soft bottom  (200-2000m) | continental slope, hard bottom  (200-2000m) | deep benthic, soft bottom  (>2000m) | deep benthic, hard bottom  (>2000m) | seamounts | shallow pelagic (<60m) | deep pelagic (>60m) |
| nutrient pollution (runoff) | 1.6 | 1.6 | 0.4 | 1.8 | 1.9 | 1.8 | 2.1 | 0.4 | 1.6 | 1.4 | 2 | 0 | 1.4 | 1.7 | 2 | 0.6 | 1.3 | 0 | 0 | 1.2 | 0 |
| organic chemical pollution (runoff) | 2.1 | 2.8 | 0.1 | 1.4 | 1.7 | 1.2 | 1 | 1 | 2.2 | 2.8 | 1.2 | 2.2 | 1.4 | 0 | 2 | 0.2 | 1.7 | 0 | 0 | 1.9 | 1.6 |
| light pollution | 1.4 | 1.4 | 2 | 0.9 | 1.8 | 1 | 0.5 | 0.5 | 0.7 | 1 | 0.5 | 0 | 0 | 0 | 0 | 0 | 0 | 0 | 0 | 0.4 | 0 |
| direct human | 2.8 | 2.2 | 2.7 | 3.3 | 1.6 | 2.3 | 2.5 | 1.6 | 2.5 | 3 | 2 | 2.6 | 1.1 | 2.9 | 0 | 0 | 1.6 | 0 | 0 | 0.9 | 0 |
| commercial fishing: demersal destructive | 1.2 | 1.4 | 0.2 | 0 | 1 | 1.2 | 0.2 | 1.5 | 2.7 | 3.1 | 2.1 | 0 | 3 | 3.1 | 3.2 | 2.8 | 2.3 | 3 | 3.5 | 2.1 | 0.8 |
| commercial fishing: demersal nondestructive high bycatch | 0.8 | 1.9 | 0.9 | 0.9 | 1 | 1.6 | 1.1 | 2.1 | 2.9 | 0.7 | 2.1 | 0 | 2 | 3.2 | 2.3 | 2.4 | 2 | 0 | 0 | 1.6 | 0 |
| commercial fishing: demersal nondestructive low bycatch | 0.6 | 1.4 | 0.7 | 0.7 | 0.8 | 1.2 | 0.8 | 1.6 | 2.2 | 0.5 | 1.6 | 0 | 1.5 | 2.4 | 1.8 | 1.8 | 1.5 | 0 | 0 | 1.2 | 0 |
| commercial fishing: pelagic high bycatch | 0.9 | 0 | 0.1 | 0 | 0.5 | 0.5 | 0 | 0 | 2.6 | 0 | 0 | 0 | 1.1 | 2.8 | 0.2 | 0 | 1.6 | 0 | 0 | 3 | 2.2 |
| commercial fishing: pelagic low bycatch | 0 | 0 | 0 | 0 | 0.4 | 0.7 | 0 | 0 | 2.6 | 0 | 0.6 | 0 | 0.8 | 2.8 | 0.2 | 0 | 0.5 | 0 | 0 | 2.2 | 0.6 |
| artisanal fishing | 1.3 | 0.4 | 0.7 | 1.7 | 0.6 | 2.3 | 0.3 | 0.8 | 2.2 | 1 | 0 | 0 | 0.9 | 1.9 | 0 | 0.4 | 0.3 | 0 | 0.9 | 1 | 0 |
| SST | 2.8 | 1.4 | 0.6 | 2.4 | 1.4 | 2.8 | 2.1 | 2 | 1.9 | 0.8 | 0.5 | 3.8 | 2.5 | 2.9 | 2.3 | 0.9 | 2.5 | 1.5 | 1.8 | 3.3 | 2.3 |
| OA | 0.9 | 1 | 0 | 1.2 | 1.3 | 1.1 | 1.4 | 0 | 1.1 | 0.7 | 0.1 | 0 | 1.7 | 2.5 | 2.1 | 1.6 | 2.2 | 2.7 | 2.7 | 1.8 | 0 |
| sea level rise | 2.5 | 1.9 | 2.1 | 3 | 3.1 | 2.4 | 2.6 | 1.6 | 1.5 | 1.8 | 2.2 | 0 | 0 | 0 | 0 | 0 | 0 | 0 | 0 | 0 | 0 |
| Shipping | 0.3 | 1.9 | 1.9 | 2 | 1.4 | 1.5 | 1.9 | 0 | 1.4 | 0 | 0.3 | 0 | 1.7 | 0.9 | 0.1 | 1 | 0.9 | 0 | 0 | 1.9 | 0 |

**Data availability**

Data from these analyses is available from KNB (Recent pace of change in human impact on the world's ocean: Cumulative impacts. Knowledge Network for Biocomplexity. doi:10.5063/F12B8WBS). Scripts for data processing are available at https://github.com/OHI-Science/impact_acceleration

# Supplementary Results

***Fig S6. Annual change in cumulative human impacts***

*Annual change in CHI estimated using a linear regression model from 2003 to 2013 for each raster cell.*


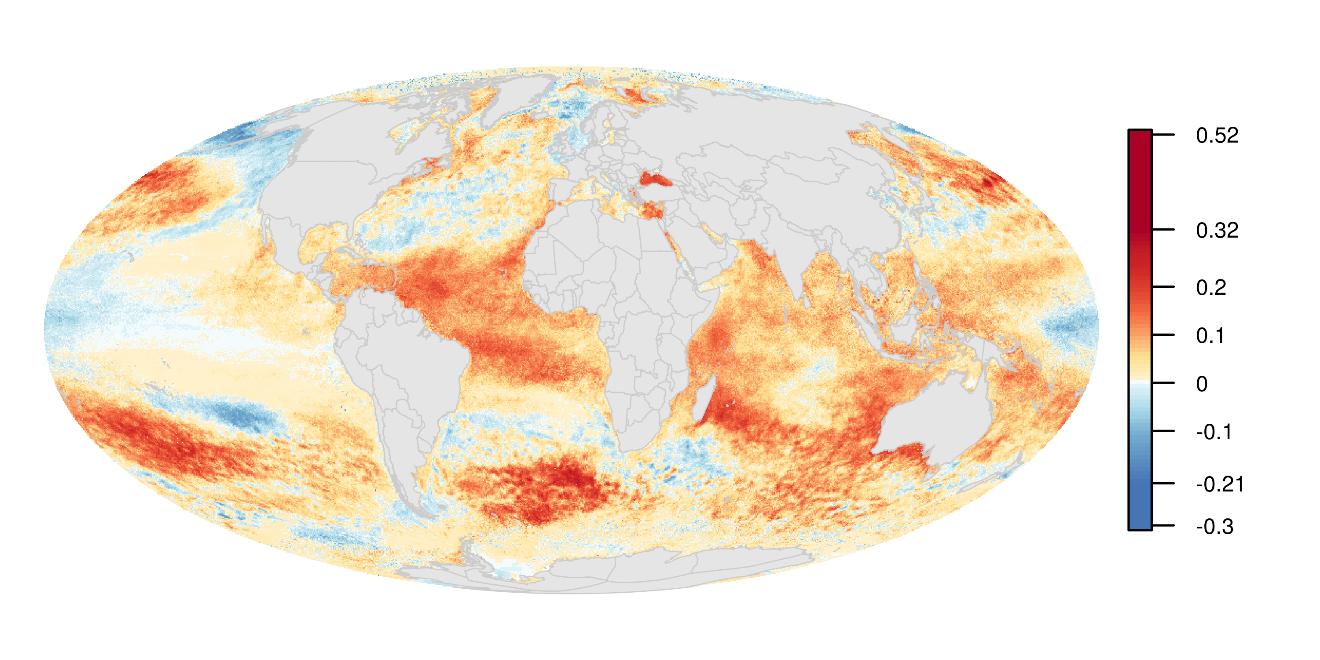


***Fig S7. Categories of annual change in cumulative impacts***

*Categories of annual change in CHI based on linear model estimates of slope from CHI values from 2003 to 2013. Areas are identified as decreasing CHI (slope < 0), and increasing CHI when: >0, >0.1, and >0.15.*


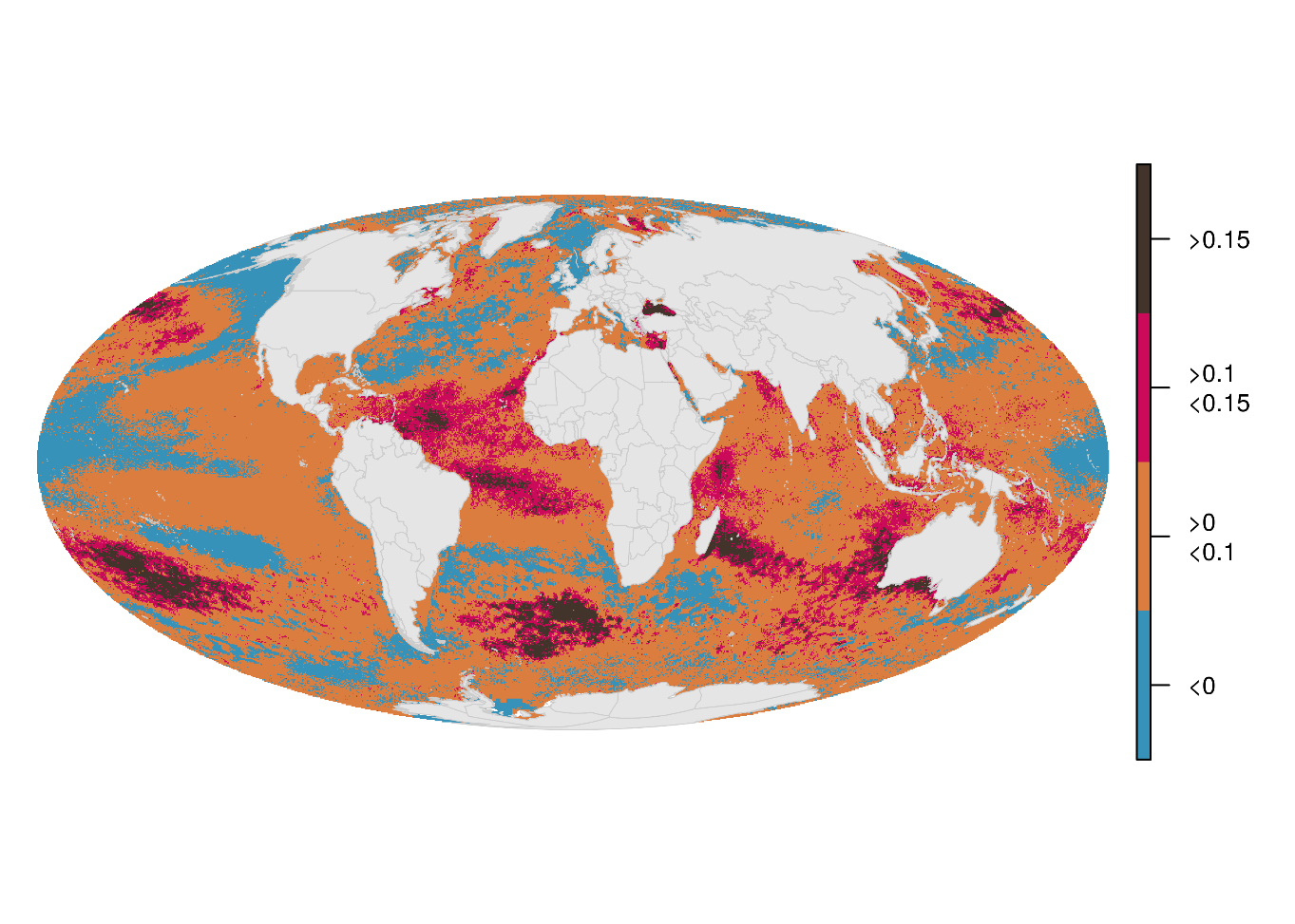


***Fig S8. CHI from 2008 to 2013***

*Cumulative human impact maps from 2003 to 2013.*


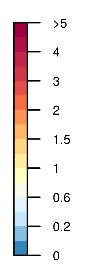

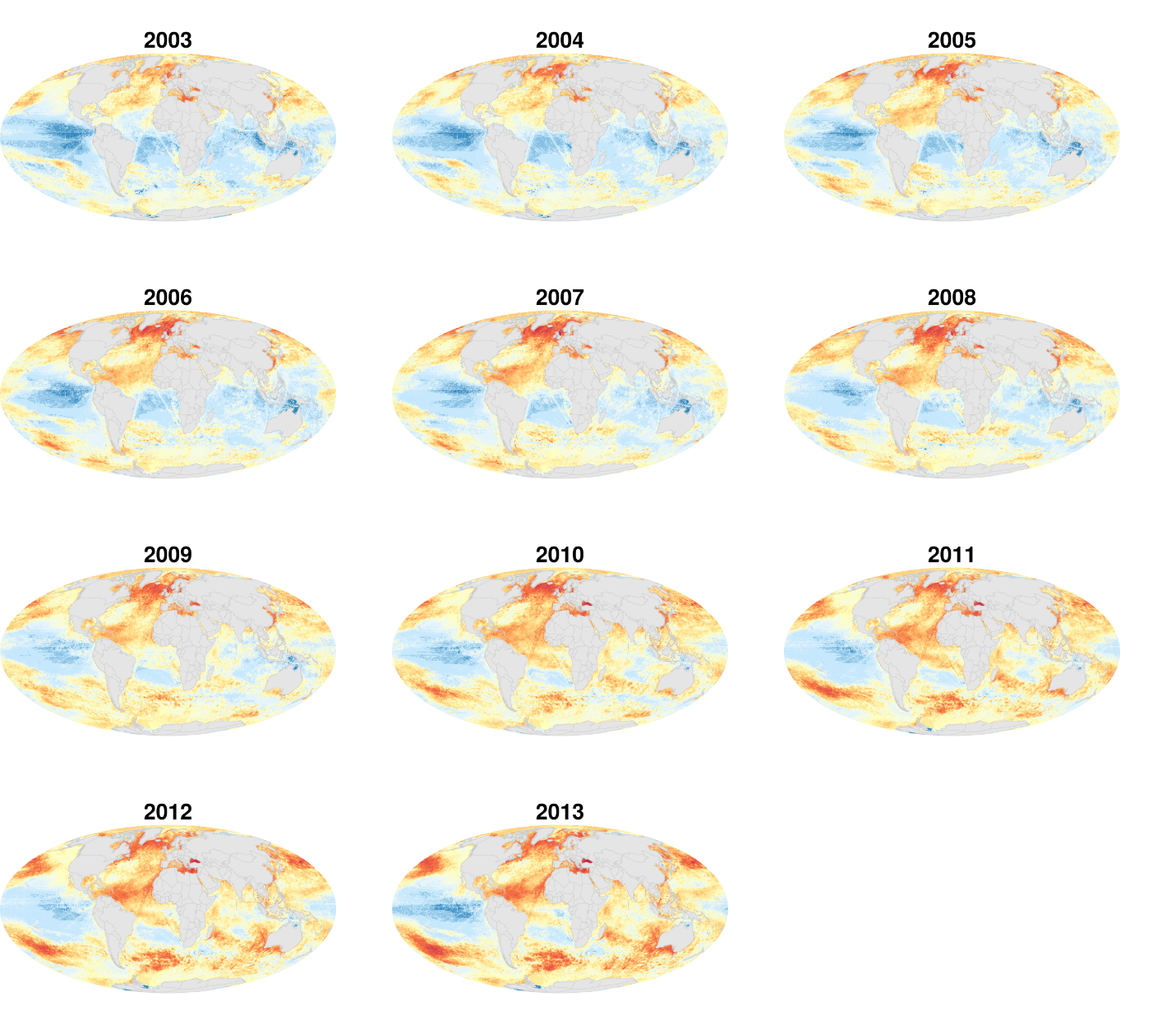


***Fig S9. SST influence on cumulative impact and trend***

*Sea surface temperature was, by far, the largest driver of global cumulative impacts (top; 2013 data) and annual change in cumulative impacts (middle; linear model of change in CHI from 2003-2013). However, most of the ocean is experiencing increasing impacts, even when SST is excluded (bottom, red indicates increasing CHI, blue decreasing CHI, and white no statistically significant pattern, P > 0.05). Left figures exclude SST and right figures include SST.*


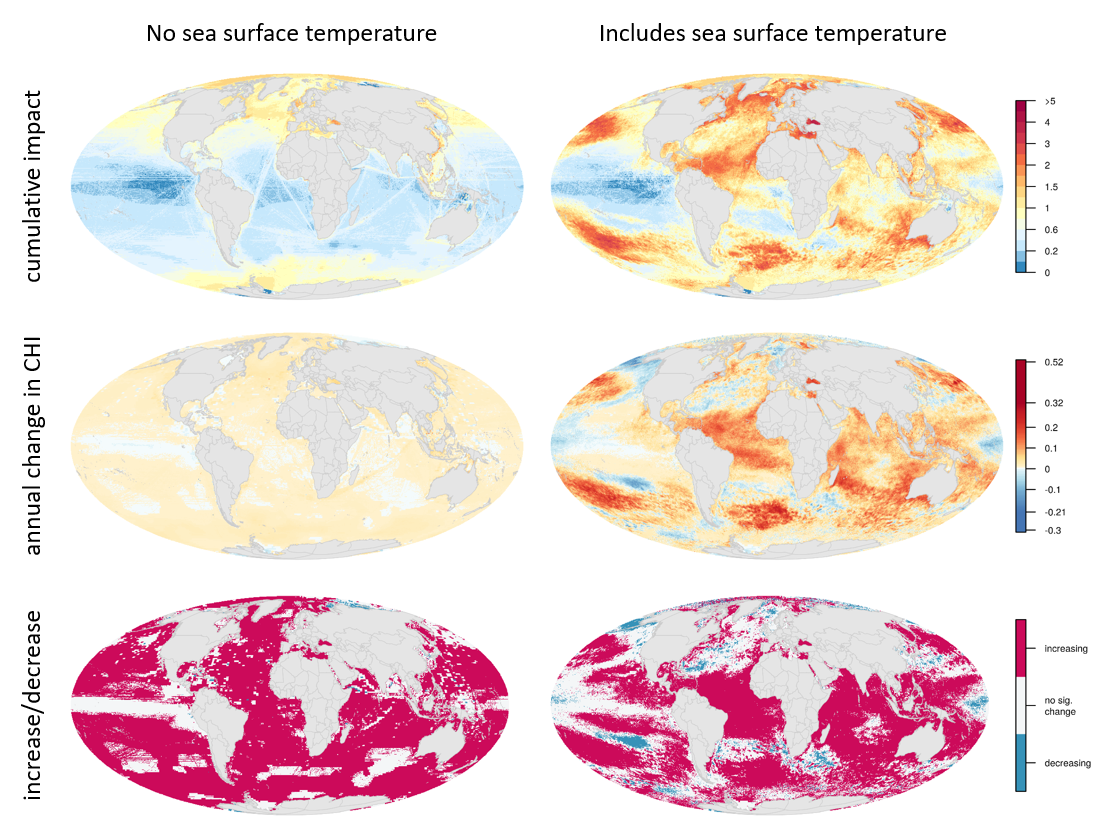


***Fig S10. Impact of SST from 2003-2017***

*The following are the SST impacts from 2003-2017. CHI estimates only include data up to 2013, during which time the SST impact was relaxed in some regions. These data indicate these regions of low impact do not persist beyond 2013.*


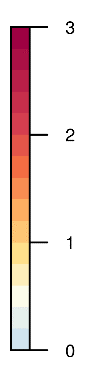

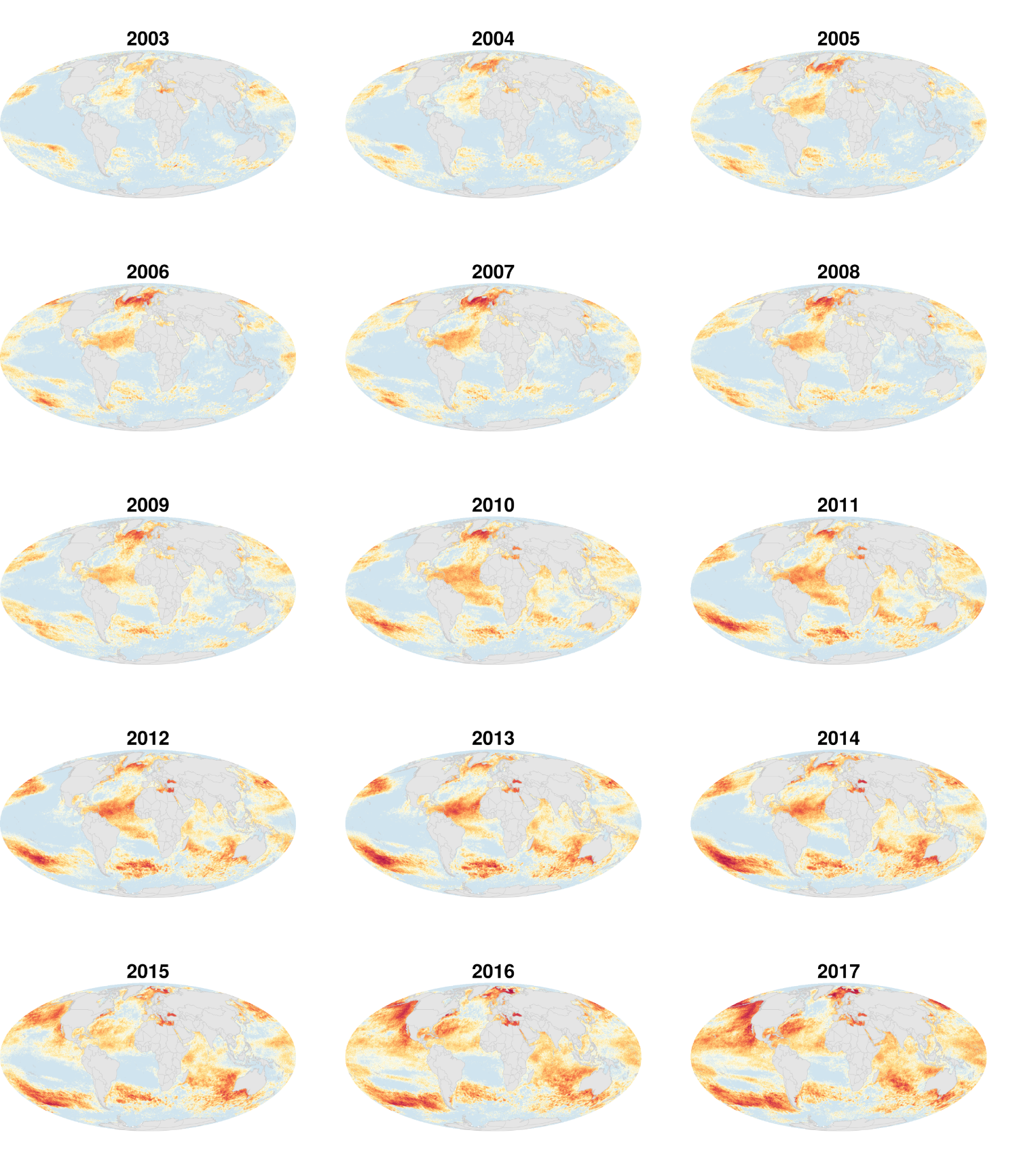


***Fig S11. Annual change in impacts for countries excluding SST***

*Annual change in impacts for 3nm offshore area for 220 countries. Sea surface temperature is excluded to explore the influence of the remaining impacts. SST was a large driver of coastal change, but other impacts were also important.*


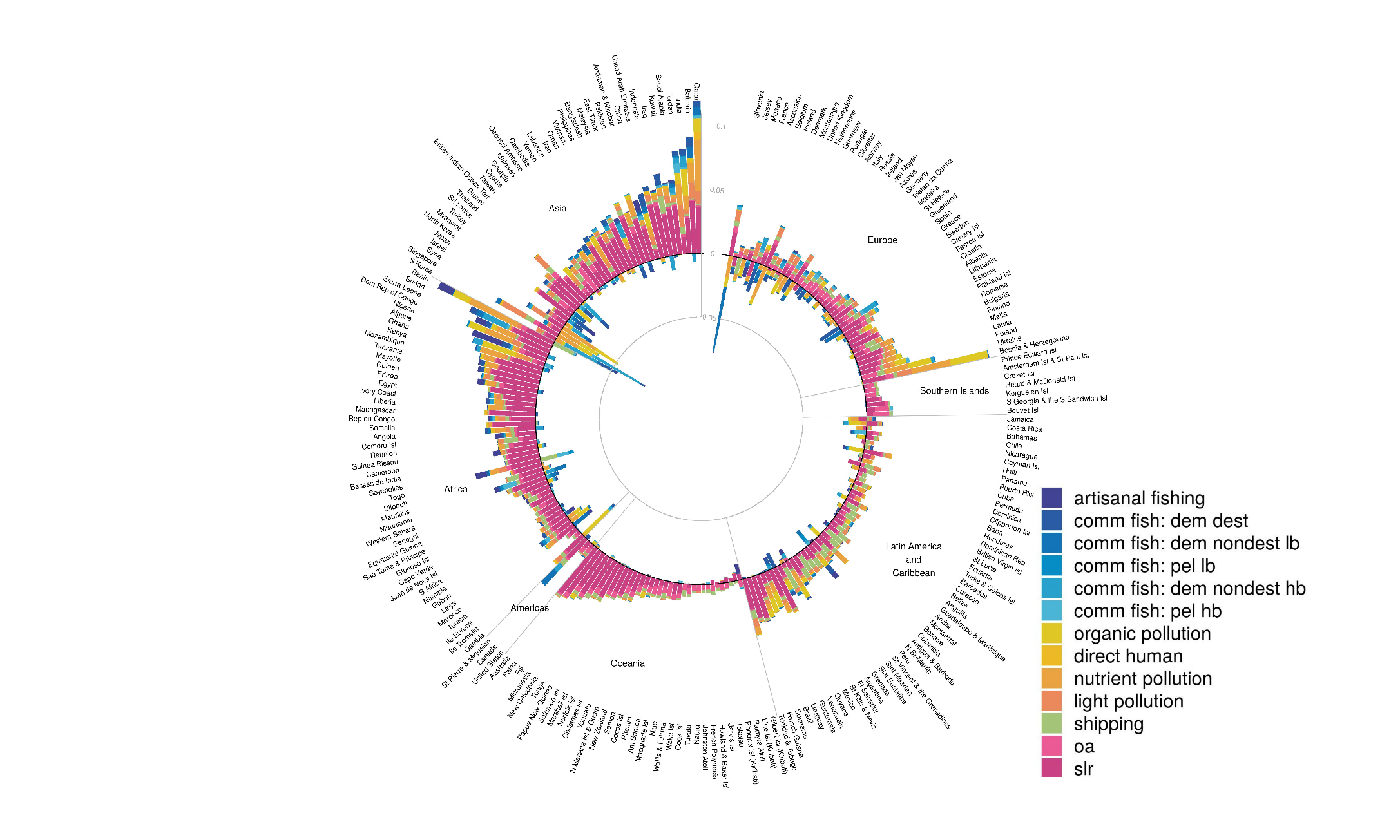


***Fig S12. Country annual change in cumulative impacts vs. cumulative impacts without SST***

*Comparison of annual change in cumulative impacts when sea surface is included vs. excluded. Most countries have increasing cumulative impacts even when sea surface temperature is excluded, but the magnitude of change is much smaller. Red solid line indicates 1:1 relationship.*


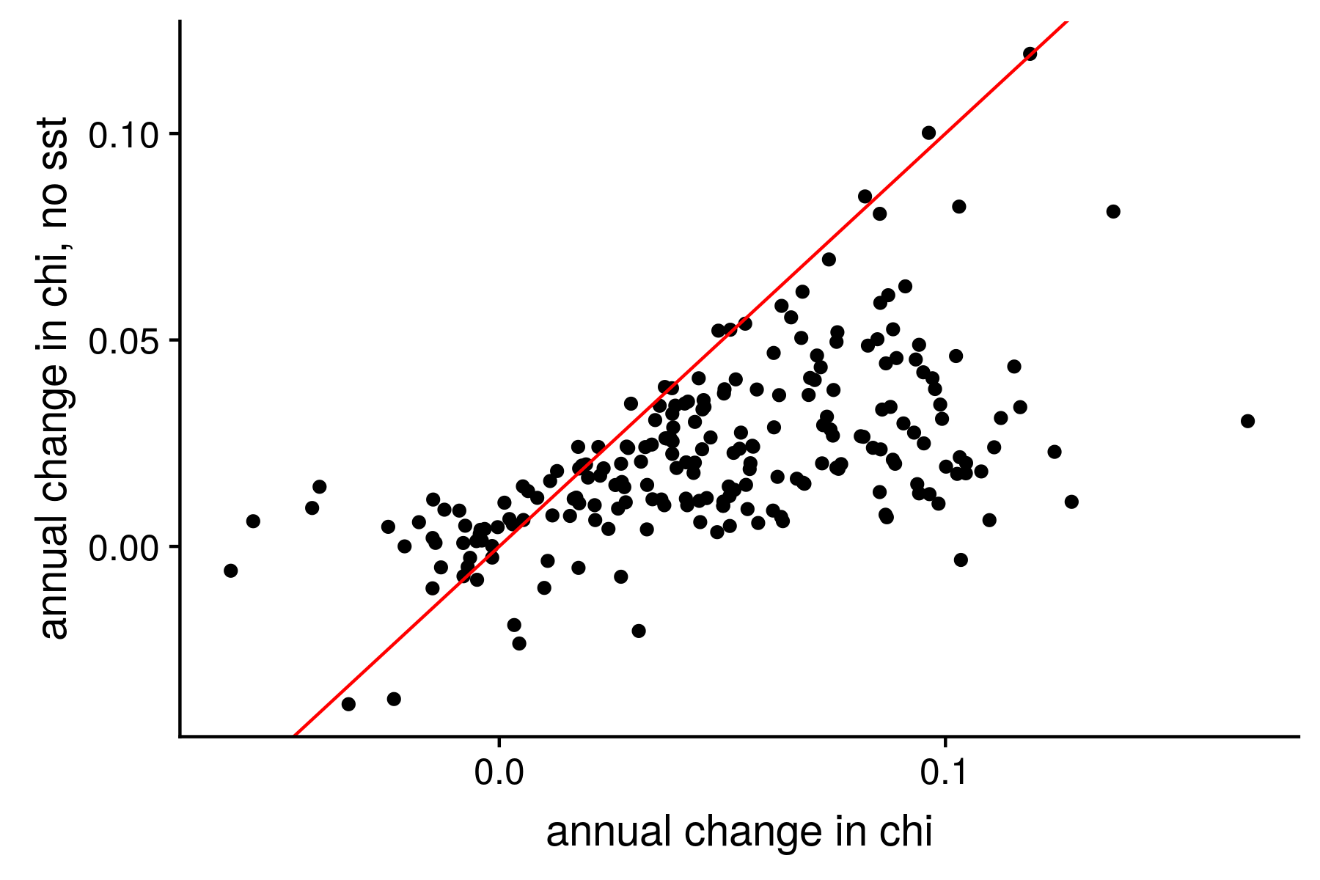


***Fig S13. Individual impacts***

*Global maps of the 14 impacts from 2013. Impacts are separated by major groups: climate change, shipping, land-based, and fishing (commercial and artisanal). Color scales are independent of one another so relative spatial variation can be visualized.*

*Climate change*


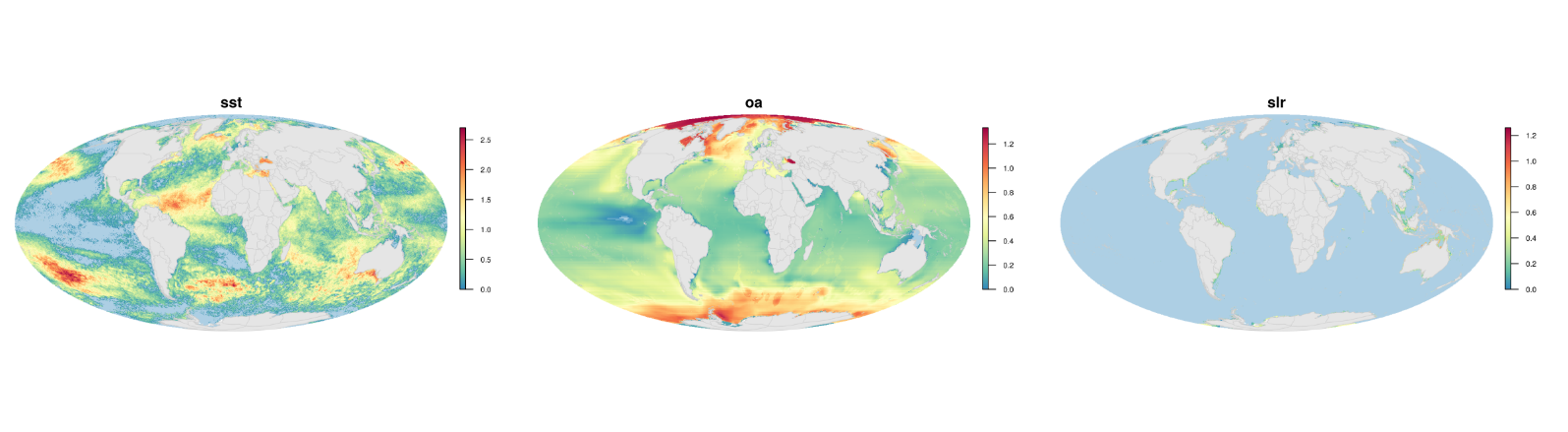


*Shipping*


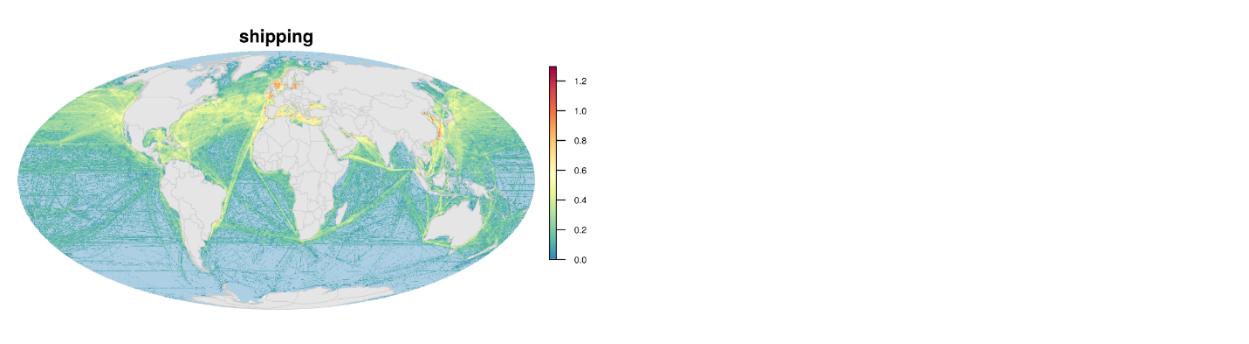


*Land-based*


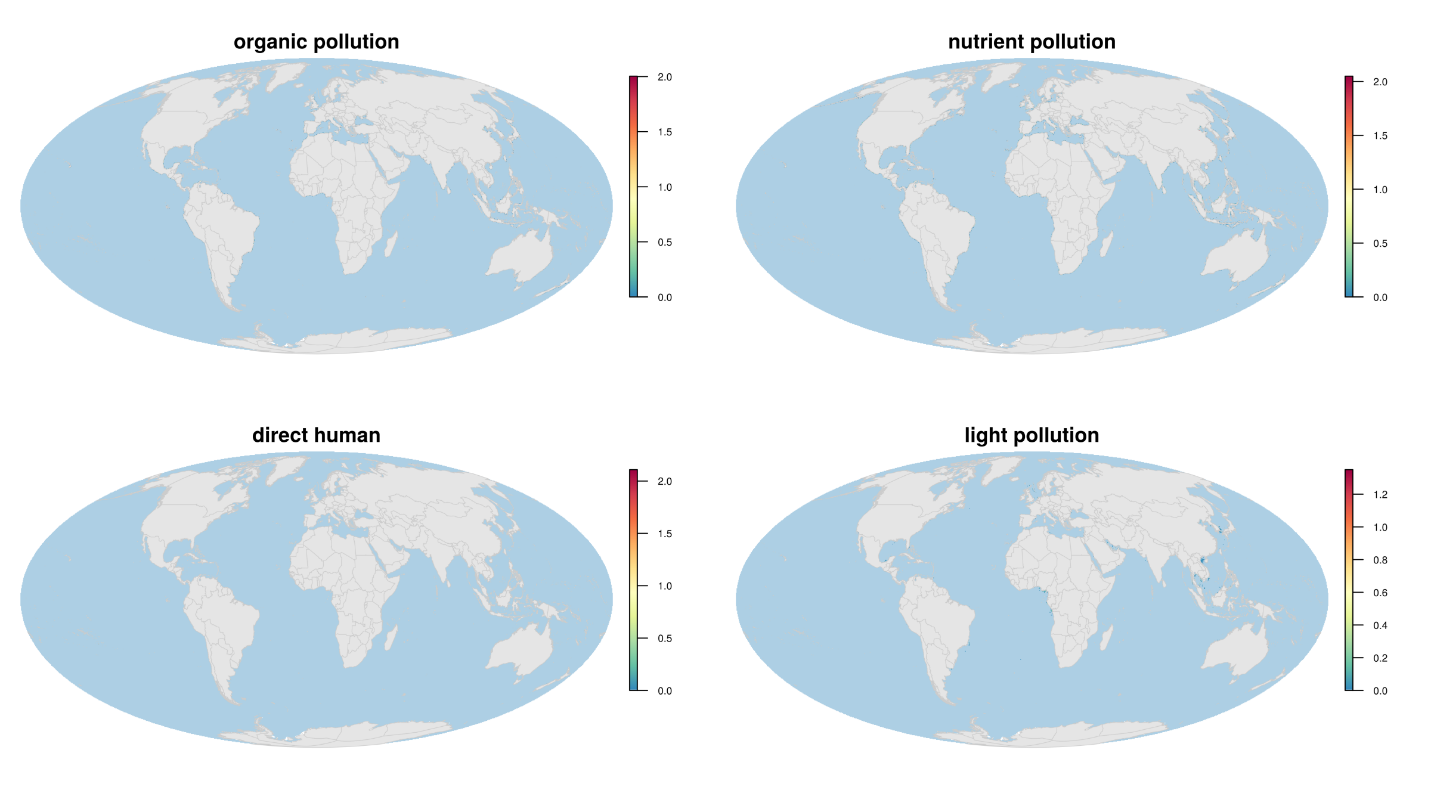


*Fishing, Fig S13 continued*


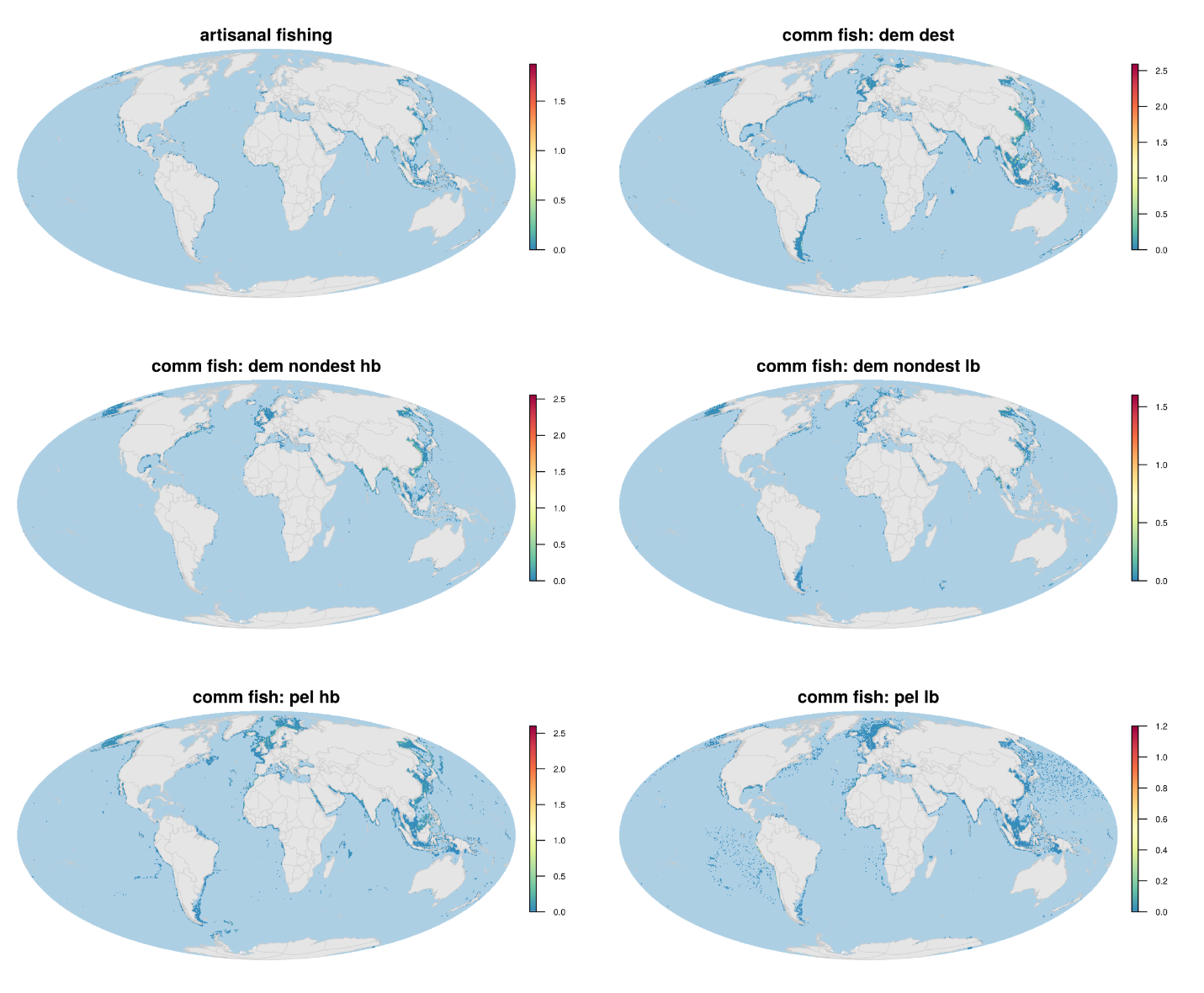


***Fig S14. Individual impact trends***

*Global maps of the change in impact from 2003 to 2013 for the 14 impacts. Impacts are separated by major groups: climate change, shipping, land-based, and fishing (commercial and artisanal). Color scales are independent of one another so relative spatial variation can be visualized.*

*Climate change*


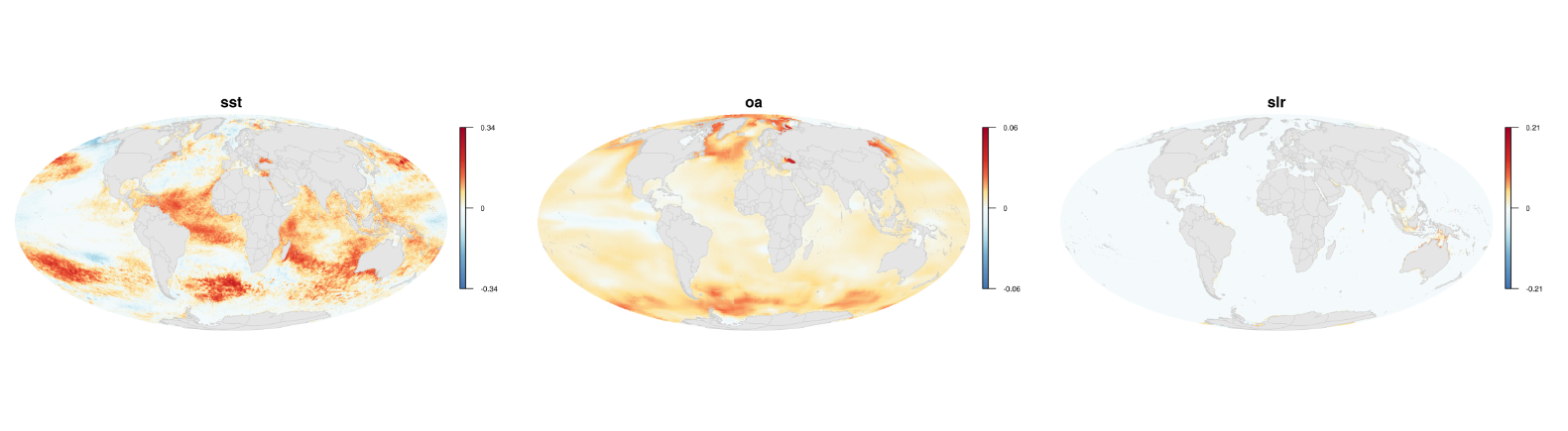


*Shipping*


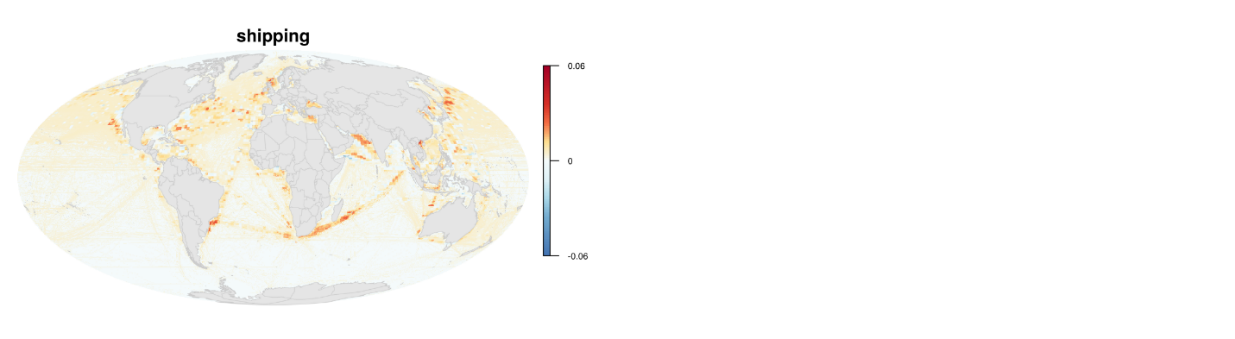


*Land-based*


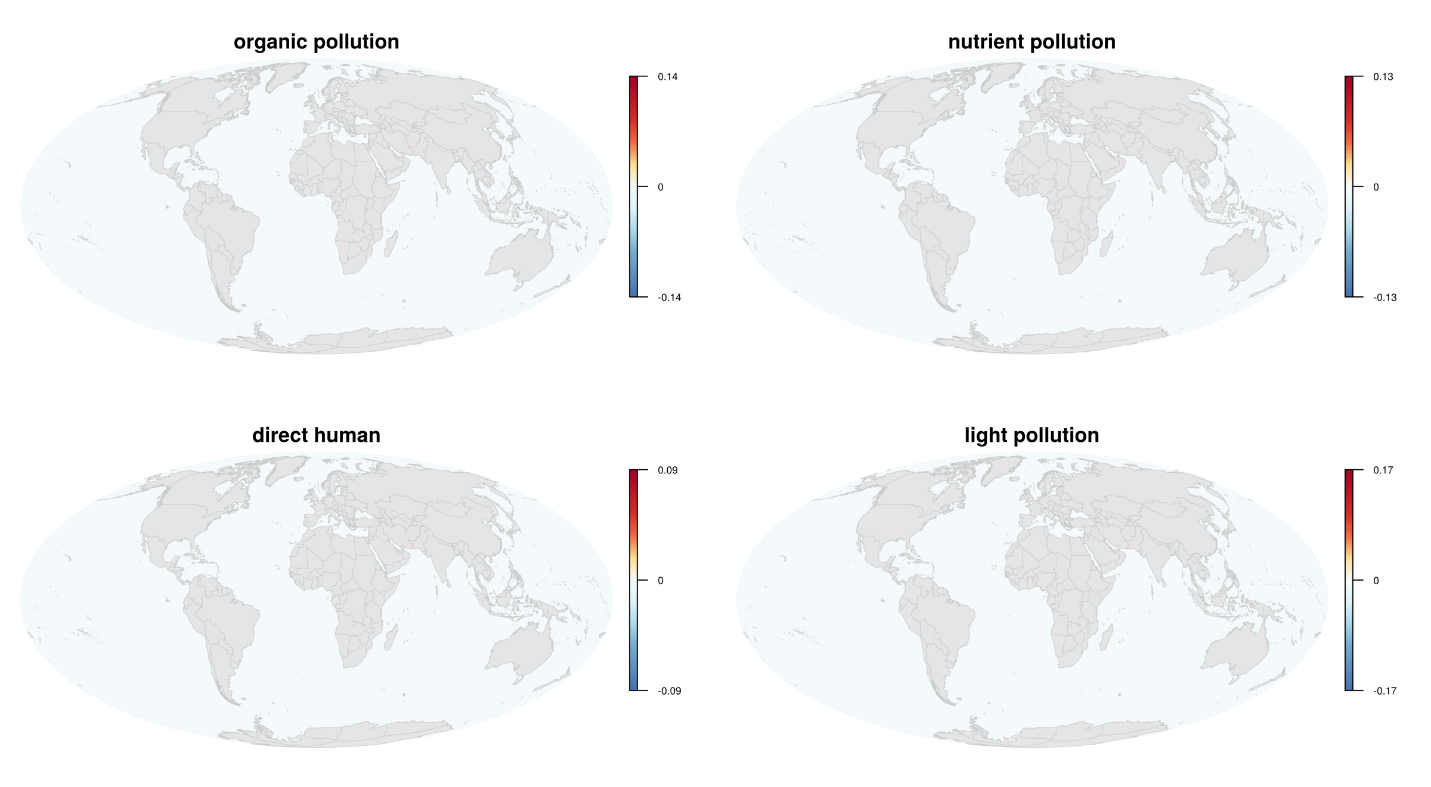


*Fishing, Fig S14 continued*


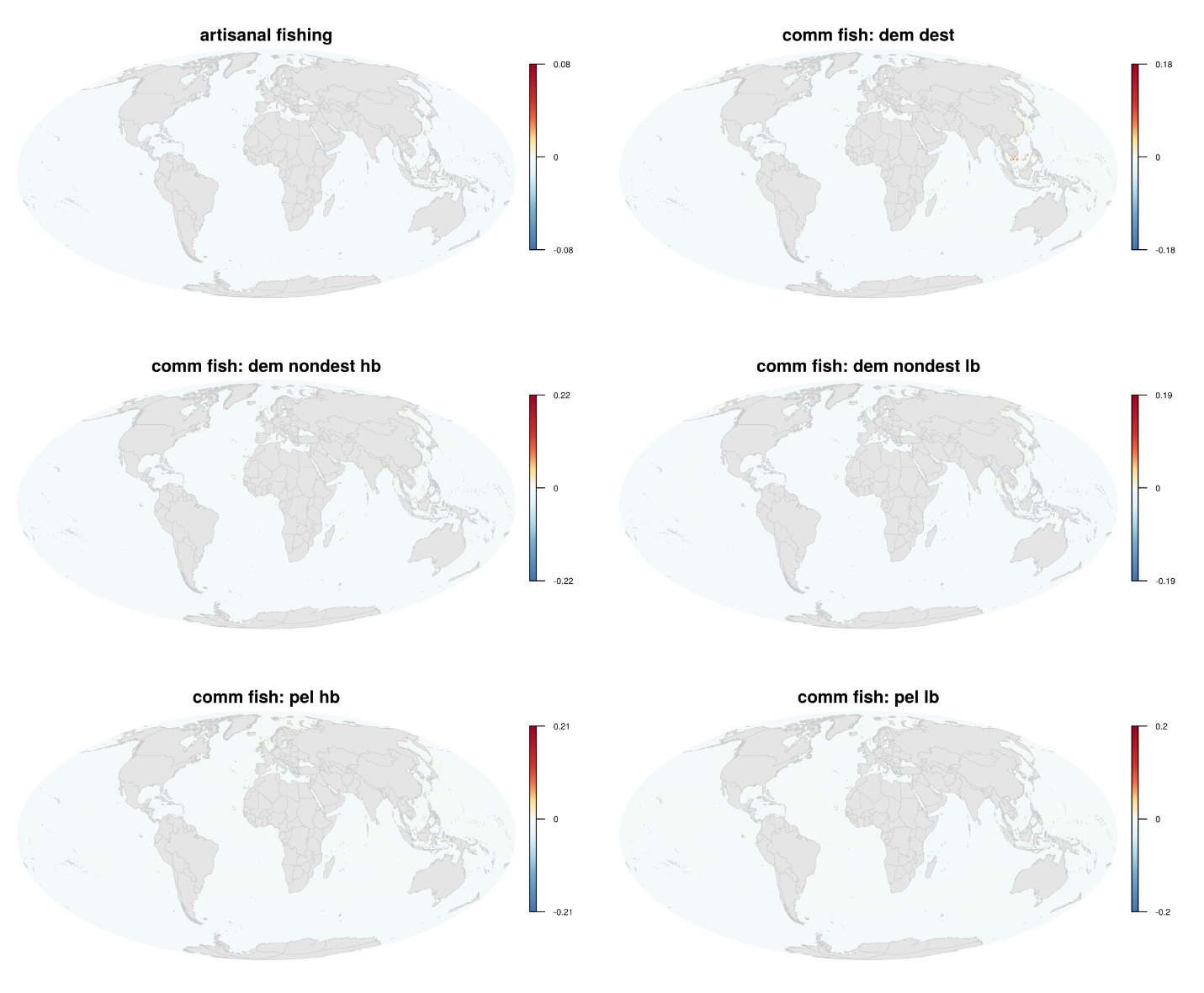


***Table S6. Global Cumulative Human Impact quantiles***

*We used quantiles to summarize global cumulative human impact data from 2003 to 2013.*

|  | **Quantiles** | | | | | |
| --- | --- | --- | --- | --- | --- | --- |
|  | *1%* | *5%* | *50%* | *95%* | *99%* | *99.99%* |
| **2003** | 0.082457 | 0.166866 | 0.592813 | 1.44663 | 1.936713 | 4.704243 |
| **2004** | 0.096577 | 0.176082 | 0.619646 | 1.475407 | 2.083218 | 4.815662 |
| **2005** | 0.109925 | 0.194055 | 0.674531 | 1.595639 | 2.252259 | 4.762044 |
| **2006** | 0.107687 | 0.203861 | 0.69875 | 1.670761 | 2.388034 | 5.016926 |
| **2007** | 0.134114 | 0.222762 | 0.728158 | 1.741522 | 2.444863 | 4.957622 |
| **2008** | 0.151883 | 0.243719 | 0.751063 | 1.761163 | 2.427743 | 4.940749 |
| **2009** | 0.173357 | 0.263123 | 0.8513 | 1.713693 | 2.294227 | 5.027513 |
| **2010** | 0.17316 | 0.267486 | 0.925238 | 1.831248 | 2.443694 | 5.205469 |
| **2011** | 0.176071 | 0.272087 | 0.958501 | 1.912712 | 2.408138 | 5.21495 |
| **2012** | 0.183967 | 0.280612 | 0.967734 | 2.017507 | 2.497396 | 5.332443 |
| **2013** | 0.122014 | 0.248735 | 1.013239 | 2.147712 | 2.65472 | 5.316513 |

***Table S7. Country and global trends in Cumulative Human Impacts***

*Summary of trends in Cumulative Human Impacts for near-coastal areas, within 3nm, of countries and territorial regions (N=220). For comparison, overall global trends are provided for the entire ocean and within 3nm of coastline.*

|  | **Country data CHI trends** | | | **Average global trends** | |
| --- | --- | --- | --- | --- | --- |
|  | *Increase (N)* | *Decrease (N)* | *Increase (%)* | *3nm* | *global* |
| **fish: dem. dest.** | 96 | 124 | 0.44 | -0.00047 | -3.00E-05 |
| **fish: dem. nondest high bycatch** | 97 | 123 | 0.44 | -0.00028 | -1.00E-05 |
| **fish: dem. nondest low bycatch** | 121 | 99 | 0.55 | -9.00E-05 | 1.00E-05 |
| **fish: pelagic low bycatch** | 107 | 113 | 0.49 | 3.00E-05 | <1.00E-05 |
| **fish: pelagic high bycatch** | 90 | 130 | 0.41 | 5.00E-05 | 2.00E-05 |
| **artisanal fisheries** | 97 | 123 | 0.44 | 0.00013 | 1.00E-05 |
| **direct human** | 155 | 65 | 0.70 | 3.00E-04 | <1.00E-05 |
| **organic pollution** | 78 | 142 | 0.35 | 0.00039 | 1.00E-05 |
| **nutrient pollution** | 97 | 123 | 0.44 | 0.00066 | 2.00E-05 |
| **light pollution** | 198 | 22 | 0.90 | 0.00121 | 2.00E-05 |
| **Shipping** | 202 | 18 | 0.92 | 0.00168 | 0.00253 |
| **ocean acidification** | 218 | 2 | 0.99 | 0.00372 | 0.00675 |
| **sea surface temperature** | 173 | 47 | 0.79 | 0.0146 | 0.03245 |
| **sea level rise** | 197 | 23 | 0.90 | 0.01531 | 0.00059 |
| **Cumulative Human Impacts** | **190** | **30** | **0.86** | **0.03724** | **0.04234** |

***Table S8. Mean of Cumulative Human Impact trends for each country.***

*Average trends in Cumulative Human Impacts for near-coastal areas, within 3nm, of countries and territorial regions (N=220). Decreasing trends indicate improving conditions.*

| **Country** | **CHI trend** |
| --- | --- |
| Gilbert Islands (Kiribati) | -0.06016 |
| Nauru | -0.05514 |
| Jan Mayen | -0.04193 |
| Pitcairn | -0.04027 |
| South Korea | -0.03377 |
| Howland Island and Baker Island | -0.02488 |
| Slovenia | -0.02359 |
| Palmyra Atoll | -0.02121 |
| Johnston Atoll | -0.018 |
| Jersey | -0.01495 |
| United Kingdom | -0.01493 |
| Crozet Islands | -0.01481 |
| Phoenix Islands (Kiribati) | -0.01429 |
| Line Islands (Kiribati) | -0.01306 |
| United States | -0.01229 |
| Ireland | -0.00895 |
| Denmark | -0.00807 |
| France | -0.00802 |
| Norway | -0.00763 |
| Bahamas | -0.00708 |
| Belgium | -0.00654 |
| North Korea | -0.00514 |
| Japan | -0.00497 |
| Netherlands | -0.00438 |
| Tokelau | -0.0042 |
| Montenegro | -0.00391 |
| Guernsey | -0.00327 |
| Chile | -0.00159 |
| Iceland | -0.00158 |
| Jarvis Island | -0.00036 |
| Germany | 0.00116 |
| Tuvalu | 0.002271 |
| French Polynesia | 0.003033 |
| Israel | 0.003352 |
| Singapore | 0.004484 |
| Greenland | 0.005293 |
| Bermuda | 0.005417 |
| Macquarie Island | 0.00645 |
| Turks and Caicos Islands | 0.008514 |
| Monaco | 0.01009 |
| Gambia | 0.010858 |
| Sweden | 0.011401 |
| Russia | 0.011909 |
| Croatia | 0.012952 |
| Italy | 0.015846 |
| Libya | 0.016688 |
| Canada | 0.017267 |
| Falkland Islands | 0.017685 |
| Costa Rica | 0.017762 |
| Namibia | 0.017898 |
| Azores | 0.017916 |
| Albania | 0.018539 |
| Lithuania | 0.019444 |
| New Zealand | 0.01989 |
| Tunisia | 0.021384 |
| Cuba | 0.021507 |
| Djibouti | 0.022209 |
| Faeroe Islands | 0.02261 |
| South Africa | 0.023385 |
| Portugal | 0.024471 |
| Gabon | 0.026023 |
| Cook Islands | 0.026627 |
| Jamaica | 0.02728 |
| Peru | 0.027301 |
| Samoa | 0.027475 |
| American Samoa | 0.028028 |
| Ecuador | 0.028346 |
| Argentina | 0.028553 |
| Estonia | 0.028901 |
| Iran | 0.029524 |
| Syria | 0.03127 |
| Senegal | 0.031767 |
| Norfolk Island | 0.03273 |
| Cayman Islands | 0.033087 |
| Spain | 0.03314 |
| El Salvador | 0.034181 |
| Myanmar | 0.034342 |
| Finland | 0.034943 |
| Yemen | 0.035954 |
| Madeira | 0.03634 |
| Ile Europa | 0.036991 |
| Eritrea | 0.037105 |
| Mexico | 0.037251 |
| Taiwan | 0.037624 |
| Marshall Islands | 0.038294 |
| Poland | 0.038713 |
| Western Sahara | 0.038772 |
| Uruguay | 0.038786 |
| Togo | 0.03885 |
| Cameroon | 0.038988 |
| Latvia | 0.03945 |
| Sri Lanka | 0.039738 |
| Oman | 0.041552 |
| Wallis and Futuna | 0.041824 |
| Bouvet Island | 0.041886 |
| Prince Edward Islands | 0.042126 |
| Brazil | 0.04229 |
| Kerguelen Islands | 0.043542 |
| Equatorial Guinea | 0.043835 |
| Guatemala | 0.043853 |
| Bangladesh | 0.044701 |
| Morocco | 0.044821 |
| Panama | 0.045037 |
| Mauritania | 0.045461 |
| Angola | 0.045542 |
| Somalia | 0.045795 |
| Cambodia | 0.045994 |
| Saint Helena | 0.046491 |
| Guyana | 0.047355 |
| Nicaragua | 0.048836 |
| China | 0.049109 |
| Wake Island | 0.050177 |
| British Virgin Islands | 0.050196 |
| Amsterdam Island and Saint Paul Island | 0.050231 |
| Republique du Congo | 0.050355 |
| Ivory Coast | 0.050482 |
| Cocos Islands | 0.051429 |
| Heard and McDonald Islands | 0.051615 |
| Gibraltar | 0.05167 |
| United Arab Emirates | 0.051777 |
| Thailand | 0.05251 |
| Belize | 0.052706 |
| Vietnam | 0.053 |
| Brunei | 0.053855 |
| Bassas da India | 0.054166 |
| Iraq | 0.055121 |
| Anguilla | 0.055303 |
| Dominican Republic | 0.05564 |
| Colombia | 0.056122 |
| Sao Tome and Principe | 0.056292 |
| British Indian Ocean Territory | 0.056721 |
| Romania | 0.056899 |
| Liberia | 0.057725 |
| Haiti | 0.058028 |
| Honduras | 0.061352 |
| Pakistan | 0.0615 |
| Guinea Bissau | 0.061571 |
| Northern Mariana Islands and Guam | 0.062356 |
| Suriname | 0.062719 |
| Clipperton Island | 0.063168 |
| Saudi Arabia | 0.063268 |
| Puerto Rico and Virgin Islands of the United States | 0.063552 |
| Kuwait | 0.065406 |
| Canary Islands | 0.066687 |
| Ghana | 0.067684 |
| Democratic Republic of the Congo | 0.067961 |
| Greece | 0.068096 |
| Turkey | 0.06839 |
| French Guiana | 0.069339 |
| Malaysia | 0.069664 |
| Micronesia | 0.070694 |
| Australia | 0.071214 |
| Guinea | 0.072036 |
| South Georgia and the South Sandwich Islands | 0.072341 |
| Bulgaria | 0.07259 |
| Malta | 0.073435 |
| Sudan | 0.073883 |
| Georgia | 0.07423 |
| Venezuela | 0.074774 |
| Madagascar | 0.074909 |
| Juan de Nova Island | 0.075561 |
| Mozambique | 0.075581 |
| Algeria | 0.075779 |
| Antigua and Barbuda | 0.076036 |
| Northern Saint-Martin | 0.076629 |
| Solomon Islands | 0.081015 |
| Cyprus | 0.08162 |
| Bahrain | 0.081973 |
| Tanzania | 0.082616 |
| Christmas Island | 0.083739 |
| Kenya | 0.084749 |
| Niue | 0.085259 |
| Benin | 0.085283 |
| Ukraine | 0.085396 |
| Vanuatu | 0.085469 |
| Papua New Guinea | 0.085808 |
| Saba | 0.086574 |
| Trinidad and Tobago | 0.086651 |
| Dominica | 0.086864 |
| Nigeria | 0.087177 |
| New Caledonia | 0.087681 |
| Sint Maarten | 0.088207 |
| Indonesia | 0.088253 |
| Glorioso Islands | 0.088718 |
| Saint Pierre and Miquelon | 0.089022 |
| Maldives | 0.090584 |
| Sierra Leone | 0.091001 |
| Seychelles | 0.09296 |
| Mayotte | 0.093347 |
| Guadeloupe and Martinique | 0.093673 |
| Curacao | 0.094084 |
| Andaman and Nicobar | 0.094086 |
| East Timor | 0.095027 |
| Saint Kitts and Nevis | 0.095155 |
| Bosnia and Herzegovina | 0.096268 |
| Barbados | 0.096418 |
| Philippines | 0.09708 |
| Egypt | 0.097663 |
| Saint Lucia | 0.098444 |
| Lebanon | 0.098825 |
| Comoro Islands | 0.09923 |
| Cape Verde | 0.100128 |
| Palau | 0.102364 |
| Aruba | 0.102616 |
| India | 0.103059 |
| Sint Eustatius | 0.103199 |
| Ascension | 0.103463 |
| Montserrat | 0.104567 |
| Saint Vincent and the Grenadines | 0.104622 |
| Bonaire | 0.108021 |
| Ile Tromelin | 0.109853 |
| Mauritius | 0.110914 |
| Oecussi Ambeno | 0.112423 |
| Fiji | 0.115409 |
| Tonga | 0.116753 |
| Qatar | 0.118967 |
| Grenada | 0.124449 |
| Tristan da Cunha | 0.128275 |
| Jordan | 0.13763 |
| Reunion | 0.167763 |

# References

AVISO (2016). Satellite Alimetry Data: Mean sea level rise. <https://www.aviso.altimetry.fr/en/data/products/sea-surface-height-products/global/msla-mean-climatology.html>

Behrenfeld, M.J., and Falkowski, P.G. (1997). Photosynthetic rates derived from satellite-based chlorophyll concentration. Limnology and Oceanography 42, 1–20.

Center for International Earth Science Information Network - CIESIN - Columbia University (2017). Gridded Population of the World, Version 4 (GPWv4): Population Density Adjusted to Match 2015 Revision UN WPP Country Totals. Palisades, NY: NASA Socioeconomic Data and Applications Center (SEDAC). <https://doi.org/10.7927/H49884ZR>

Cavalieri, D.J., C.L. Parkinson, P. Gloersen, and H.J. Zwally. 1996, updated yearly. Sea ice concentrations from Nimbus-7 SMMR and DMSP SSM/I-SSMIS Passive Microwave Data, Version 1. Boulder, Colorado, USA. NASA National Snow and Ice Data Center Distributed Active Archive Center. doi: <https://doi.org/10.5067/8GQ8LZQVL0VL>. Download: <http://nsidc.org/data/NSIDC-0051>, July 3 2018.

Channan, S., K. Collins, and W. R. Emanuel (2014). Global mosaics of the standard MODIS land cover type data. University of Maryland and the Pacific Northwest National Laboratory, College Park, Maryland, USA.

Elvidge, C.D., Ziskin, D., Baugh, K.E., Tuttle, B.T., Ghosh, T., Pack, D.W., Erwin, E.H., and Zhizhin, M. (2009). A Fifteen Year Record of Global Natural Gas Flaring Derived from Satellite Data. Energies 2, 595–622.

Feely, R., Doney, S., and Cooley, S. (2009). Ocean acidification: present conditions and future changes in a high-CO2 world. Oceanography 22, 36–47.

Frazier, M., Longo, C., and Halpern, B.S. (2016). Mapping uncertainty due to missing data in the global Ocean Health Index. PLOS ONE 11, e0160377.

Friedl, M.A., D. Sulla-Menashe, B. Tan, A. Schneider, N. Ramankutty, A. Sibley and X. Huang (2010), MODIS Collection 5 global land cover: Algorithm refinements and characterization of new datasets, 2001-2012, Collection 5.1 IGBP Land Cover, Boston University, Boston, MA, USA. <http://www.landcover.org/data/lc/>

Green, E.P., and Short, F.T. (2003). World Atlas of Seagrasses (UNEP-WCMC). <https://www.unep-wcmc.org/resources-and-data/world-atlas-of-seagrasses>

Halpern, B.S., Walbridge, S., Selkoe, K.A., Kappel, C.V., Micheli, F., D’Agrosa, C., Bruno, J.F., Casey, K.S., Ebert, C., Fox, H.E., et al. (2008). A Global Map of Human Impact on Marine Ecosystems. Science 319, 948–952.

Halpern, B.S., Frazier, M., Potapenko, J., Casey, K.S., Koenig, K., Longo, C., Lowndes, J.S., Rockwood, R.C., Selig, E.R., Selkoe, K.A., et al. (2015). Spatial and temporal changes in cumulative human impacts on the world’s ocean. Nat Commun 6, 7615.

Jenson, S.K., and Domingue, J.O. (1988). Extracting Topographic Structure from Digital Elevation Data for Geographic Information System Analysis. Photogrammetric Engineering and Remote Sensing. 54: 1593-1600. <https://www.asprs.org/wp-content/uploads/pers/1988journal/nov/1988_nov_1593-1600.pdf>

Kitchingman, A., and Lai, S. (2004). Seamounts : biodiversity and fisheries (Vancouver, B.C.).

Pauly, D., and Zeller, D. (2015). Sea around us concepts, design and data. seaaroundus.org

Saha, K., Zhao, X., Zhang, H., Casey, K.S.. Zhang, D., Zhang, Y., Baker-Yeboah, S., Relph, J.M., Krishnan, A., Ryan, T. (2018). The Coral Reef Temperature Anomaly Database (CoRTAD) Version 6 - Global, 4 km Sea Surface Temperature and Related Thermal Stress Metrics for 1982 to 2017. NOAA National Centers for Environmental Information. Dataset. August 21, 2018.

Selig, E.R., Casey, K.S., and Bruno, J.F. (2010). New insights into global patterns of ocean temperature anomalies: implications for coral reef health and management. Global Ecology and Biogeography 19, 397–411.

Spalding, M., Blasco, F., and Field, C. (1997). World Mangrove Atlas (UNEP-WCMC). https://www.unep-wcmc.org/world-mangrove-atlas-1997

Spalding, M.D., Ravilious, C., and Green, E.P. (2001). World Atlas of Coral Reefs (UNEP-WCMC). <https://www.unep-wcmc.org/resources-and-data/world-atlas-of-coral-reefs-2001>

Steneck, R., Graham, M.H., Bourque, B.J., Corbett, D., Erlandson, J.M., Estes, J.A., and Tegner, M.J. (2002). Kelp Forest Ecosystems: Biodiversity, Stability, Resilience and Future. *29*, 436.

Tournadre, J. (2014). Anthropogenic pressure on the open ocean: The growth of ship traffic revealed by altimeter data analysis. Geophysical Research Letters 41, 7924–7932.

United Nations (2016). FAO Statistics Division - Inputs. <http://faostat3.fao.org/browse/R/*/E>

USGS (2004). Shuttle Radar Topography Mission, 30 Arc Second, SRTM30 Digital Elevation Model (DEM), Global Land Cover Facility, University of Maryland, College Park, Maryland, February 2000. <http://glcf.umd.edu/data/srtm/>

Wallbridge, S. (2013). Assessing ship movements using volunteered geographic information. University of California, Santa Barbara. Masters Thesis. <https://4326.us/thesis/walbridge-masters-thesis.pdf>

Watson, R.A., and Tidd, A. (2018). Mapping nearly a century and a half of global marine fishing: 1869–2015. Marine Policy 93, 171–177.

Watson, R.A. (2018). Global Fisheries Landings V3.0. <http://metadata.imas.utas.edu.au/geonetwork/srv/eng/metadata.show?uuid=ff1274e1-c0ab-411b-a8a2-5a12eb27f2c0>.
